# METTL8 orchestrates T_FH_ cell differentiation through m³C-mediated TCF1 regulation in infection and cancer models

**DOI:** 10.1172/JCI199747

**Published:** 2026-11-16

**Authors:** Jiaxi Song, Dan Cui, Jiabao Wang, Xuewei Qi, Jiaxin Ma, Haoyu Yu, Qing Liu, Jing Yang, Tengfei Hu, Xu Han, Handong Ma, Rentao Yu, Ying Dai, Xiaona Zhang, Qunfa Cao, Xiaoyuan Song, Bofeng Li, Lilin Ye, Hua Wang, Qizhao Huang, Jia Li, Fengyin Li

**Affiliations:** 1 State Key Laboratory of Immune Response and Immunotherapy, School of Basic Medical Sciences, Division of Life Sciences and Medicine, University of Science and Technology of China, Hefei, China.; 2 Guangzhou National Laboratory, Guangzhou, China.; 3 Hefei National Research Center for Physical Sciences at the Microscale, Ministry of Education Key Laboratory of Cellular Dynamics, Chinese Academy of Sciences Key Laboratory of Brain Function and Disease, School of Life Sciences, Division of Life Sciences and Medicine, University of Science and Technology of China, Hefei, China.; 4 Department of Dermatology, The First Affiliated Hospital of Chongqing Medical University, Chongqing, China.; 5 Department of Oncology, The First Affiliated Hospital of Anhui Medical University, Hefei, China.; 6 National Engineering Center for Biochip at Shanghai, Shanghai, China.; 7 Shanghai Biochip Corporation, Shanghai, China.; 8 Institute of Immunological Innovation and Translation, Chongqing Medical University, Chongqing, China.; 9 Changping Laboratory, Beijing, China.; 10 Key Laboratory of Anti-Inflammatory and Immune Medicine, Ministry of Education, Hefei, China.; 11 State Key Laboratory of Respiratory Disease, National Clinical Research Center for Respiratory Disease, National Center for Respiratory Medicine, Joint International Research Laboratory of Respiratory Health, Guangdong Basic Research Center of Excellence for Respiratory Medicine, Guangzhou Institute of Respiratory Health, the First Affiliated Hospital of Guangzhou Medical University, Guangzhou, China.; 12 China-Portugal Artificial Intelligence and Public Health Technologies Joint Laboratory, Guangdong-Hong Kong-Macao Joint Laboratory of Respiratory Infectious Diseases, Guangdong Provincial Key Laboratory of Respiratory Disease Research, Guangzhou Medical University. Guangzhou, China.

**Keywords:** Cell biology, Oncology, Immunotherapy

## Abstract

T follicular helper (T_FH_) cells are critical for humoral immunity against infections and correlate with immunotherapy efficacy, whereas immune checkpoint inhibitor therapy can drive their aberrant expansion, underscoring the need to understand the regulators controlling T_FH_ cell fate. However, the epitranscriptomic regulation of their differentiation remains poorly understood. Here, we identify the 3-methylcytosine (m³C) methyltransferase METTL8 as a key regulator of T_FH_ cell differentiation in both viral infection and cancer. METTL8 was preferentially expressed in CXCL13^hi^ T_FH_ cells, and its deficiency impaired T_FH_ differentiation and germinal center responses in the lymphocytic choriomeningitis virus infection model and following anti–PD-1 immunotherapy in tumor models. Mechanistically, METTL8 exerted dual functions: it bound and stabilized *Tcf7* mRNA through m³C modification, and it interacted with TCF1 protein to regulate chromatin accessibility and 3D genome organization at key T_FH_-associated loci, including *Cxcr5* and *Il6ra*. Notably, ginkgolic acid treatment suppressed T_FH_ responses without compromising the antitumor efficacy of anti–PD-1 therapy, an effect associated with METTL8. Collectively, our findings identify METTL8 as an epitranscriptomic regulator linked to RNA modification, TCF1-associated transcriptional control, and chromatin architecture during T_FH_ differentiation and suggest that modulation of this pathway may provide a strategy to tune T_FH_-driven immune responses.

## Abstract 2

METTL8 regulates T_FH_ immune cells in infection and cancer models; inhibiting it doesn’t reduce anti‑PD‑1 cancer treatment efficacy.

## Introduction

T follicular helper (T_FH_) cells, a specialized CD4^+^ T cell subset, serve as coordinators of germinal center (GC) responses, providing crucial help to B cells for survival, proliferation, class switching, and affinity maturation to generate effective antibodies (1–3). These functions are pivotal for protection against infectious diseases and cancer. In viral infections, such as lymphocytic choriomeningitis virus (LCMV) and influenza, T_FH_ cells are indispensable for the generation of high‑affinity neutralizing antibodies and the establishment of long‑term humoral memory (4, 5). In the tumor microenvironment, T_FH_ cells contribute to the formation and function of tertiary lymphoid structures and are associated with favorable clinical outcomes and enhanced responses to immune checkpoint blockade (6, 7). However, the benefits of T_FH_ cells are also double‑edged in immune checkpoint inhibitor (ICI) therapy. Because they express high levels of PD‑1, anti–PD-1 therapy directly promotes T_FH_ expansion and differentiation, leading to excessive B cell help, autoantibody production, and tissue‑directed inflammation via IL‑21 and CXCL13 (8). Clinically, T_FH_ expansion correlates with immune‑related adverse event development, positioning T_FH_ cells as a key link between ICI efficacy and autoimmune toxicity (9, 10). Therefore, identifying a target that suppresses aberrant T_FH_ expansion without compromising the antitumor efficacy of ICIs is of critical importance.

The differentiation and function of T_FH_ cells are tightly controlled at multiple levels, including transcriptional regulation, signaling pathways, and epigenetic modifications. The transcription factor TCF1 (encoded by *Tcf7*) has been established as a master regulator of T_FH_ cell differentiation, promoting the expression of key T_FH_-associated genes such as *Cxcr5*, *Pdcd1*, and *Bcl6* (11–13). Additionally, the chromatin landscape and 3D genome organization play crucial roles in establishing cell-type–specific transcriptional programs during T_FH_ differentiation (14, 15). However, the epitranscriptomic mechanisms that modulate T_FH_ cell differentiation and function remain poorly understood.

RNA modifications have emerged as critical posttranscriptional regulators of gene expression in immune cells. Among these, 3-methylcytosine (m³C) is a recently characterized modification that occurs on both mitochondrial and cytosolic tRNAs, as well as on mRNAs (16–19). METTL8 has been identified as a methyltransferase that catalyzes m³C modification on specific tRNAs and regulates mitochondrial protein translation and respiration (16–18). However, whether METTL8 plays a role in CD4^+^ T cell differentiation, particularly in the T_FH_ lineage, has not been explored.

In this study, we identified METTL8 as a regulator of T_FH_ cell differentiation in viral infection and tumor immunotherapy models. We found that METTL8 is highly expressed in inflammatory CD4^+^CXCL13^+^ T_FH_ cells. Using the acute LCMV infection model and MC38 and B16F10 tumor models, we demonstrated that METTL8-mediated m³C modification is important for T_FH_ differentiation and GC responses in both contexts. Mechanistically, our data support a model in which METTL8 stabilizes *Tcf7* mRNA in an m³C-associated manner and cooperates with TCF1 protein to regulate genome-specific chromatin loops and enhance chromatin accessibility at key T_FH_ loci. Furthermore, we show that GA suppresses T_FH_ responses in a METTL8-associated manner without compromising the antitumor efficacy of anti–PD-1 therapy. Collectively, our findings identify METTL8 as a key epitranscriptomic regulator of T_FH_ cell differentiation, providing potential avenues for modulating T_FH_ responses in immunological disorders.

## Results

### METTL8 is highly expressed in CXCL13^hi^ CD4^+^ T cells.

Although ICIs have achieved therapeutic success in various malignancies, their administration drives marked expansion of CD4^+^ T cells and can elicit autoimmune‑like tissue manifestations, with the skin being one of the most frequently affected sites. To characterize the CD4^+^ T cell populations induced by anti–PD‑1 therapy, we collected skin samples from patients with tumors who had developed dermatitis after anti–PD-1 treatment and performed spatial transcriptome sequencing (Supplemental Figure 1A; supplemental material available online with this article; https://doi.org/10.1172/JCI199747DS1). The data revealed *CD4* expression in the skin samples (Figure 1A and Supplemental Figure 1B). As CXCL13 is expressed in CD4^+^ T cells within autoimmune-affected organs (20), we detected *CXCL13* expression in many CD4^+^ regions of the skin samples (Figure 1A and Supplemental Figure 1B) (21). *PDCD1*, the characteristic marker of GC T_FH_ cells, was also detected in tissue regions enriched for *CD4* and *CXCL13* expression (Figure 1A and Supplemental Figure 1B), suggesting the presence of T_FH_-like CD4^+^ cells in these areas. To further explore CXCL13 expression in the T_FH_ cells generated by anti–PD-1 therapy, we treated B16F10 tumor–bearing mice with anti–PD-1 antibody and tested T_FH_ cells in their spleens and tumor-draining lymph nodes (TdLNs) (Figure 1B). Anti–PD-1 treatment significantly suppressed tumor growth (Supplemental Figure 1, C and D). Compared with IgG-treated controls, anti–PD-1–treated mice exhibited markedly increased frequencies of GC T_FH_ cells in both spleens and TdLNs (Figure 1C and Supplemental Figure 1E). Notably, anti–PD-1 treatment significantly increased the absolute number of CXCL13^hi^ GC T_FH_ cells and modestly increased their frequency (Figure 1D and Supplemental Figure 1F), and CXCL13 expression levels were substantially elevated in T_FH_ cells following anti–PD-1 treatment (Figure 1E and Supplemental Figure 1G). Collectively, these results indicate that anti–PD-1 treatment expands the population of CXCL13^hi^ T_FH_ cells.

Given that cancer immunotherapy elicits autoimmune responses characterized by expansion of CXCL13^hi^ GC T_FH_ cells, which share similar features with spontaneous autoimmune diseases, and that GC T_FH_ cells in the classic LCMV mouse infection model also express high levels of CXCL13 (Figure 1F), we sought to identify key epitranscriptomic regulators that commonly govern these cells. Therefore, we compared CD4^+^ T cells from the salivary glands of patients with Sjögren’s syndrome (SS), a spontaneous autoimmune disease, with those from healthy individuals treated as controls and performed integrative analysis with differentially expressed genes between T_FH_ and T_H_1 cells from the classical LCMV mouse infection model.

By reanalyzing published scRNA-seq data from salivary glands in healthy individuals and patients with SS (22), we identified 4 CD4^+^ T cell clusters, including proliferating CD4^+^ T cells, T_reg_ cells, CXCL13^hi^, and CXCL13^lo^ CD4^+^ T cells (Supplemental Figure 1I). To depict the characteristics of the latter 2 clusters, we analyzed the differentially expressed genes between these cells and found that CXCL13^hi^ CD4^+^ T cells highly expressed T_FH_-featured genes, such as *CXCR5*, *PDCD1*, *BCL6*, and *TCF7*, while CXCL13^lo^ CD4^+^ T cells were prone to T_H_1 cells (Figure 1G). Further analysis of differentially expressed genes revealed that these highly expressed T_FH_-associated genes were also enriched in the CD4^+^ T cells of patients with SS compared with healthy individuals (Figure 1H), suggesting that these CXCL13^hi^ T_FH_ cells increased in affected organs of the autoimmune disease. These results further support the presence of CXCL13^hi^ T_FH_-like cells with B cell helper features in autoimmune-affected tissues.

To identify factors potentially involved in T_FH_ cell development, we performed a cross-analysis of genes highly expressed in T_FH_ cells from the canonical LCMV-infected mouse model (11) and those upregulated in CXCL13^hi^ CD4^+^ T cells from patients with SS. The overlapping gene set predominantly comprised known T_FH_-associated genes, including *CXCR5*, *TCF7*, and *PDCD1*. Notably, *METTL8*, which encodes an m³C methyltransferase and has not previously been implicated in T_FH_ cell biology, was also present among the common genes (Figure 1I). These findings suggest that METTL8 may serve as a shared regulator of CXCL13^hi^ T_FH_ cells across distinct pathological and physiological contexts. Further analysis revealed that *METTL8* was highly expressed in CD4^+^ T cells of patients with SS compared with those of healthy individuals, which is consistent with the expression of *CXCL13* (Figure 1I). Thus, we hypothesize that METTL8 contributes to CXCL13^hi^ T_FH_ cell specification.

To determine whether METTL8 is expressed in T_FH_ cells following ICI treatment, we first analyzed transcriptomic data from skin samples and observed high METTL8 expression in the CD4^+^CXCL13^+^PD-1^+^ regions (Figure 2A). We then examined METTL8 expression in the CD4^+^BCL6^+^ T_FH_ cells within skin samples from patients with anti–PD-1–treated tumors. Compared with untreated controls, anti–PD-1–treated patients exhibited increased CD4^+^ T cell infiltration in the skin. Notably, these infiltrating CD4^+^ T cells expressed both BCL6 and METTL8, whereas such cells showed little or no detectable staining for these markers in untreated controls (Figure 2B and Supplemental Figure 2A). Collectively, these findings indicate that METTL8 is expressed in T_FH_ cells upon anti–PD-1 therapy.

To further validate this observation, we inoculated B16F10 cells into *Mettl8*-tdTomato-Flag reporter mice and treated them with either IgG or anti–PD-1 antibody (Figure 2C). Analysis of PD-1^hi^CXCR5^hi^ GC T_FH_ cells in the spleens and TdLNs revealed that METTL8 expression levels were higher in anti–PD-1–treated mice than in IgG-treated controls (Figure 2D and Supplemental Figure 2B). Taken together, these results support the conclusion that METTL8 is expressed in T_FH_ cells following treatment in both human and mouse samples.

To further investigate whether METTL8 is expressed preferentially in T_FH_ cells, we infected *Mettl8*-tdTomato-Flag mice with LCMV-Armstrong and analyzed them at 8 days after infection (Figure 2E). We confirmed that *Mettl8*-tdTomato was highly expressed in PD-1^hi^CXCR5^hi^ GC T_FH_ cells compared with PD-1^lo^CXCR5^+^ T_FH_ cells or CXCR5^−^ non-T_FH_ cells (Figure 2F). Considering these consistent expression trends of CXCL13 and METTL8 in both the human disease and the mouse model, we conducted further analysis that revealed that the expression of METTL8 was much higher in CXCL13^hi^ CD4^+^ T cells than in CXCL13^lo^ CD4^+^ T cells (Figure 2G). These results indicate that METTL8 is preferentially expressed in CXCL13^hi^ GC T_FH_ cells.

Further analysis revealed that *METTL8* expression was significantly higher in CD4^+^ T cells of SS patients than in those from healthy controls, a pattern consistent with *CXCL13* expression (Figure 2H). In addition, *METTL8* expression was markedly elevated in CXCL13^hi^ clusters compared with CXCL13^lo^ clusters (Figure 2I). These findings suggest that METTL8 may contribute to the specification of CXCL13^hi^ T_FH_ cells.

### METTL8 promotes T_FH_ cell differentiation.

To further explore whether METTL8 promotes T_FH_ cell generation by tumor immunotherapy, we subcutaneously inoculated B16F10 or MC38 cells into *Mettl8*^fl/fl^ *Cd4*^cre^ mice and littermate controls and then treated mice with anti–PD-1 antibody or IgG (Figure 3A). The data showed that *Mettl8* deficiency did not affect the frequency and absolute number of CD4^+^ T cells in either spleens or TdLNs (Supplemental Figure 3, A and B). CD4^+^ T cell activation was also not detectably affected by *Mettl8* deficiency (Supplemental Figure 3, C and D). However, PD-1^hi^CXCR5^hi^ and BCL6^+^CXCR5^+^ GC T_FH_ cells were decreased in *Mettl8*-deficient mice compared with littermate controls after anti–PD-1 treatment in both spleens and TdLNs (Figure 3B and Supplemental Figure 3, E and F). Thus, these data indicate that METTL8 promotes T_FH_ cell accumulation after anti–PD-1 treatment.

To further validate the effect of METTL8 on T_FH_ cells, we used the canonical LCMV-infected mouse model. *Mettl8*^fl/fl^ *Cd4*^cre^ mice were crossed with a Smarta TCR transgenic strain expressing an MHC II–restricted TCR specifically recognizing the LCMV gp61 epitope. WT and *Mettl8*^−/−^ Smarta CD4^+^ T cells were adoptively transferred into congenic recipients, followed by infection with LCMV-Armstrong (Figure 3C). In response to infection, *Mettl8*^−/−^ Smarta CD4^+^ T cells exhibited a marked decrease in PD-1^hi^CXCR5^hi^ and BCL6^+^CXCR5^+^ GC T_FH_ cells (Figure 3D). Furthermore, LCMV infection showed no difference between the frequencies and absolute numbers of CD44^hi^CD62^lo^ activated CD4^+^ T cells of *Mettl8*^fl/fl^ *Cd4*^cre^ mice and littermate controls (Supplemental Figure 4A), but GC T_FH_ cells were impaired by *Mettl8* deficiency (Supplemental Figure 4, B and C). In addition, we observed a reduction in SLAM^lo^CXCR5^+^ T_FH_ cells among monoclonal and polyclonal CD4^+^ T cells, accompanied by increased frequencies and unchanged counts of SLAM^hi^CXCR5^−^ T_H_1 cells (Supplemental Figure 4, D and E). The PD-1^lo^CXCR5^+^ T_FH_ cells were also reduced by *Mettl8* deficiency, whereas the number of CXCR5^−^ non-T_FH_ cells was not affected, although the frequency increased (Supplemental Figure 4F). Notably, the expression of caspase and Ki67 in T_FH_ cells was unaffected by *Mettl8* deficiency, suggesting that METTL8 does not affect apoptosis or proliferation of T_FH_ cells (Supplemental Figure 4G). Therefore, these results indicate that *Mettl8* deficiency impairs T_FH_ cell differentiation.

To further substantiate the requirement of METTL8 in T_FH_ cell differentiation, we enforced METTL8 expression in *Mettl8*^−/−^ or control (Ctrl) Smarta CD4^+^ T cells using pMSCV-IRES-GFP retroviruses. GFP^+^ and GFP^−^ cells were sorted and cotransferred into recipient mice at a 1:1 ratio (Figure 3E and Supplemental Figure 4H). Mice were infected with LCMV-Armstrong, and at the 8 day postinfection (dpi) time point, the ratio of GFP^+^ to GFP^−^ cells remained unaltered (Supplemental Figure 4I), indicating comparable survival and proliferation of transduced and nontransduced cells. Relative to empty vector–GFP (EV-GFP) control, forced expression of METTL8 in Ctrl Smarta cells significantly increased PD-1^hi^CXCR5^hi^ and BCL6^+^CXCR5^+^ GC T_FH_ cells (Figure 3F). Importantly, ectopic METTL8 expression in *Mettl8*^−/−^ Smarta cells significantly rescued the reduction in GC T_FH_ cells caused by *Mettl8* deficiency (Figure 3F). Moreover, SLAM^lo^CXCR5^+^ T_FH_ cells were restored, and the frequency of SLAM^hi^CXCR5^−^ T_H_1 cells was decreased by METTL8 overexpression in *Mettl8*^−/−^ Smarta cells (Supplemental Figure 4J). These data further support a requirement for METTL8 in T_FH_ cell differentiation.

### METTL8 promotes T_FH_ cell function.

To assess the impact of *Mettl8*-deficient T_FH_ cells on tumor progression, we monitored tumor growth and survival in MC38 or B16F10 tumor–bearing *Mettl8*^fl/fl^ *Cd4*^cre^ mice and littermate controls. Antitumor responses, with or without anti–PD-1 treatment, were comparable between genotypes in these models (Figure 4, A and B, and Supplemental Figure 5, A and B). Given the essential role of T_FH_ cells in supporting B cell responses, we next examined the GC response in TdLNs. Upon anti–PD-1 treatment, *Mettl8* deficiency led to a reduction in GC B cells and plasma cells within TdLNs (Figure 4C and Supplemental Figure 5C). Collectively, these data indicate that *Mettl8* deficiency impairs the GC response in TdLNs following anti–PD-1 therapy, without affecting tumor progression.

To examine the functional requirement of METTL8 in the infection model, we transferred *Mettl8*^−/−^ or Ctrl Smarta CD4^+^ T cells to *Bcl6*^fl/fl^ *Cd4*^cre^ mice, which were deficient in endogenous GC T_FH_ cells (Figure 4D) (23). *Mettl8*-deficient PD-1^hi^CXCR5^hi^ and BCL6^+^CXCR5^+^ GC T_FH_ cells were dramatically reduced at 14 dpi (Supplemental Figure 5, D and E). Accordingly, recipients of *Mettl8*^−/−^ Smarta cells exhibited reduced Fas^+^GL7^+^ GC B cells and CD138^+^IgD^lo^ plasma cells (Figure 4, E and F). Similarly, direct LCMV-Armstrong infection of *Mettl8*^fl/fl^ *Cd4*^cre^ mice resulted in decreased GC B cells and plasma cells at 8 dpi compared with littermate controls (Supplemental Figure 5, F and G), and GC formation was impaired (Figure 4G). Consistently, LCMV-specific IgG in the serum of *Bcl6*^fl/fl^ *Cd4*^cre^ recipients was significantly reduced by *Mettl8* deficiency (Figure 4H). Furthermore, *Mettl8* deficiency led to elevated viral loads (Figure 4I). Although LCMV-Armstrong infection normally induces only mild hepatitis in mice (24), representative H&E images showed more prominent inflammatory infiltrates in portal tracts and intralobular regions (Figure 4J). Histological examination further revealed that follicle-associated regions were diminished in the spleens of *Mettl8*^−/−^ Smarta cell–transferred mice (Supplemental Figure 5H). These data support an important role for METTL8 in GC responses.

### METTL8 promotes T_FH_ cell accumulation and maintenance.

As T_H_1 and T_FH_ fate determination occurs promptly following the activation of CD4^+^ T cells, we investigated the potential involvement of METTL8 in the T_FH_ fate decision process. Initially, we assessed METTL8 expression in activated CD4^+^ T cells in vitro, observing heightened expression with prolonged stimulation (Supplemental Figure 6A). To further explore METTL8 expression, we utilized *Mettl8*-tdTomato Smarta CD4^+^ T cells labeled with cell-trace violet and adoptively transferred them into congenic recipients, followed by LCMV-Armstrong infection (Supplemental Figure 6B). Harvesting cells at the 40 and 60 h postinfection (hpi) time point, corresponding to the initiation of the first division at 36 hpi and the fifth division at 60 hpi (25) for Smarta CD4^+^ T cells, we examined METTL8 expression in each division from the first to the fifth (Supplemental Figure 6C). The data revealed elevated METTL8 expression during CD4^+^ T cell activation and the first division, maintaining a sustained high level despite slight decreases during subsequent divisions (Figure 5A). These results indicate that METTL8 expression is increased by CD4^+^ T cell activation during the early T_H_1-T_FH_ fate bifurcation stage, prompting us to investigate whether METTL8 plays a role during this period.

To explore the role of METTL8 in T_FH_ lineage specification, we utilized *Mettl8*^fl/fl^ *Cd4*^cre^ mice and littermate controls. Following adoptive transfer of cell-trace violet–labeled *Mettl8*^−/−^ or Ctrl Smarta CD4^+^ T cells and LCMV-Armstrong infection, both groups underwent at least 5 divisions, with the majority of cells in the fourth and fifth divisions at 60 hpi, indicating comparable division rates (Supplemental Figure 6D). These findings suggest that the loss of METTL8 does not disrupt CD4^+^ T cell activation and early division. Despite this, the reduction of T_FH_ cells upon *Mettl8* deficiency led us to infer that METTL8 may contribute to T_FH_ versus T_H_1 lineage bifurcation.

As previously reported, the combination of CD25 and CXCR5 was employed to distinguish nascent T_H_1 and T_FH_ cells in activated CD4^+^ T cells (25). In the fifth division, *Mettl8*^−/−^ CD25^lo^CXCR5^+/lo^ nascent T_FH_ cells were detected at a lower frequency compared with their WT counterparts, accompanied by an opposite change in the frequency of CD25^hi^CXCR5^−^ nascent T_H_1 cells (Supplemental Figure 6E). Additionally, CXCR5 expression was slightly but significantly reduced in *Mettl8*-deficient nascent T_FH_ cells, whereas it remained unchanged in nascent T_H_1 cells (Supplemental Figure 6E). These results suggest that METTL8 exerts modest effects on early T_FH_ specification. We therefore examined T_FH_ cells from days 4 to 7 after infection and found that the reduction in T_FH_ cells caused by *Mettl8* deficiency became progressively more pronounced over time (Figure 5B and Supplemental Figure 6F). Consequently, our findings indicate that METTL8 has a limited impact on early T_FH_ differentiation but plays a substantial role in T_FH_ accumulation and maintenance.

To unravel the underlying mechanism governing T_FH_ cell development mediated by METTL8, we conducted RNA sequencing on sorted PD-1^hi^CXCR5^hi^ GC T_FH_ cells obtained from LCMV-Armstrong–infected *Mettl8*^fl/fl^ *Cd4*^cre^ mice and their littermate controls at 8 dpi. Our analysis identified 1,597 downregulated genes and 1,469 upregulated genes in *Mettl8*-deficient GC T_FH_ cells, each exhibiting a fold change > 2 (Figure 5C). Notably, these differentially expressed genes showed enrichment in pathways related to T_FH_ cell differentiation (Figure 5D). GSEA further revealed that genes downregulated in T_FH_ cells compared with non-T_FH_ cells were enriched in *Mettl8*-deficient GC T_FH_ cells, while genes upregulated in T_FH_ cells versus non-T_FH_ cells were enriched in WT GC T_FH_ cells (Figure 5E). Among the identified upregulated and downregulated genes, those encoding transcription factors crucial for T_FH_ cell differentiation, such as *Bcl6*, *Maf*, *Tcf7*, *Cebpa, Tox*, *Id3*, *Pou2af1*, *Sostdc1*, and *Ascl2*, were consistently downregulated in *Mettl8*-deficient GC T_FH_ cells (Figure 5F and Supplemental Figure 6G). Additionally, the expression of genes defining GC T_FH_ cell characteristics, including *Cxcr5*, *Pdcd1*, *Icos*, and *Il21*, exhibited a decrease in *Mettl8*-deficient mice (Figure 5F and Supplemental Figure 6G). Consistent with our RNA-seq findings, flow cytometric analysis confirmed a significant reduction in the expression of T_FH_ cell surface markers CXCR5 and ICOS, as well as transcription factors BCL6 and TCF1 (Figure 5G). Conversely, genes upregulated in *Mettl8*-deficient GC T_FH_ cells tended to exhibit T_H_1 signatures, including *Prdm1*, *Tbx21*, *Id2*, and *Il2ra* (Figure 5F and Supplemental Figure 6G). The increased expression of T-bet was validated by flow cytometry (Figure 5G). In summary, these results indicate that METTL8 biases activated CD4^+^ T cells toward the T_FH_ program rather than the T_H_1 program.

### METTL8 facilitates m^3^C-associated regulation of Tcf7 mRNA and cooperates with TCF1-associated chromatin programs.

To elucidate the mechanisms by which METTL8-mediated m^3^C methylation modulates the T_FH_ transcriptional program, we conducted AlkAniline-seq (26) to identify distinct m^3^C modification loci between PD-1^hi^CXCR5^hi^ GC T_FH_ cells isolated from LCMV-Armstrong–infected *Mettl8*^fl/fl^ *Cd4*^cre^ mice and littermate controls. The global mapping of the m^3^C landscape revealed abundant m^3^C peaks within the coding sequence of mRNA (Supplemental Figure 7, A and B). Comparative analysis of m^3^C modification loci in *Mettl8*-deficient and WT GC T_FH_ cells identified 4,491 different sites, with 1,425 in WT T_FH_ cells and 3,066 in *Mettl8*-deficient T_FH_ cells (Supplemental Figure 7C). Significantly enriched RNA motifs flanking m³C modification loci from WT GC T_FH_ cells are presented (Supplemental Figure 7D). These data revealed AlkAniline-seq for mapping m^3^C modifications in T_FH_ cells.

Given that METTL8 is a mitochondrially localized enzyme that catalyzes m^3^C modification on tRNAs and regulates mitochondrial protein translation and respiration (16–18), we first performed tRNA m³C-seq to examine whether *Mettl8* deficiency affects tRNA modification in T_FH_ cells. However, we observed no significant differences in tRNA m³C modification between WT and *Mettl8*-deficient T_FH_ cells (Supplemental Figure 7, F and G). To further assess mitochondrial respiratory function, we performed Seahorse metabolic flux analysis in WT and *Mettl8*-deficient T_FH_ cells. No statistically significant differences were detected in the measured bulk oxygen consumption rate (OCR) and extracellular acidification rate (ECAR) parameters under the tested conditions (Supplemental Figure 7H). Collectively, these results suggest that METTL8 does not appreciably affect tRNA m³C modification or global mitochondrial metabolism in T_FH_ cells, prompting us to investigate its potential mRNA-targeted functions.

To further investigate METTL8 binding sites on mRNA, RNA-immunoprecipitation and sequencing (RIP-seq) was performed on PD-1^hi^CXCR5^hi^ GC T_FH_ cells isolated from LCMV-Armstrong–infected mice. A combined analysis of RNA-seq, m^3^C-seq, and RIP-seq revealed 94 genes commonly targeted by m^3^C and METTL8, with differential RNA expression between *Mettl8*-deficient and WT GC T_FH_ cells (Supplemental Figure 7E). To define a direct relationship between METTL8 binding and differential m³C modification, we required that a METTL8 binding site must be located within 500 bp of a differential m^3^C modification site on the same transcript (27, 28), rather than merely co-occurring anywhere on the same transcript. To evaluate the biological significance of the observed overlap, we performed a Fisher’s exact test based on the entire set of expressed genes to compare the observed number of overlapping genes against the expected value under a random distribution. Our analysis demonstrated that the overlap is statistically significant (*P* < 0.01, OR > 2.5), indicating that the intersecting genes are unlikely to arise by chance. Notably, *Tcf7* showed overlapping m^3^C-seq and RIP-seq signals (Figure 6A). To validate the regulation of METTL8 on *Tcf7*, a pMigR1-GFP-*Tcf7* vector was constructed, and retrovirus was generated to enforce TCF1 expression in Smarta cells. Strikingly, TCF1 overexpression in *Mettl8^−/−^* Smarta cells significantly restored the PD-1^hi^CXCR5^hi^ GC T_FH_ cell frequency (Figure 6, B and C). Correspondingly, the frequency of PD-1^lo^CXCR5^+^ T_FH_ cells was increased, and that of CXCR5^−^ non-T_FH_ cells was decreased by TCF1 overexpression in *Mettl8^−/−^* Smarta cells compared with *Mettl8^−/−^* Smarta cells transfected with pMigR1 EV (Supplemental Figure 7I). To test whether METTL8 regulates *Tcf7* mRNA stability, an RNA decay assay was performed using actinomycin D and showed that *Tcf7* mRNA declined faster in *Mettl8*-deficient cells than in control cells (Figure 6D), indicating that METTL8 enhances *Tcf7* mRNA stability.

Considering the genomic coordination and histone acetylation modification ability of TCF1 (29–31), we performed ATAC-seq (assay for transposase-accessible chromatin with high-throughput sequencing) on *Mettl8*^−/−^ and control PD-1^hi^CXCR5^hi^ GC T_FH_ cells and observed reduced chromatin accessibility in *Mettl8*-deficient GC T_FH_ cells (Figure 6E). The decrease in chromatin accessibility mainly occurred at the gene transcription initiation site (Supplemental Figure 7J). To investigate the cooperation of METTL8 with TCF1 in organizing chromatin architecture (32) in GC T_FH_ cells, we integrated Hi-C (high-resolution chromosome-conformation capture), TCF1 CUT&Tag, and ATAC-seq data from *Mettl8*^−/−^ and control GC T_FH_ cells with METTL8 CUT&Tag data from *Mettl8*-tdTomato GC T_FH_ cells. We also performed Hi‑C on T_H_1 cells to identify T_FH_‑specific loops affected by METTL8. The analysis identified locus-specific chromatin loops at TCF1 target genes, including *Il6ra* and *Cxcr5*, where METTL8 and TCF1 CUT&Tag signals overlapped. These regions also showed H3K27ac enrichment and increased chromatin accessibility, consistent with active regulatory elements at key GC T_FH_ genes (Figure 6F). Notably, these T_FH_‑specific loops were largely absent in T_H_1 cells, indicating that the 3D genome organization at these loci is lineage restricted (Figure 6F).

In *Mettl8*^−/−^ GC T_FH_ cells, TCF1 binding at these loci was substantially reduced, accompanied by disruption of chromatin looping and decreased chromatin accessibility. These findings suggest that METTL8 promotes TCF1-dependent chromatin organization and maintains an accessible regulatory landscape at genes essential for GC T_FH_ cell identity and function (Figure 6F). Together, these results suggest that METTL8 cooperates with TCF1-associated regulatory programs to promote T_FH_-associated gene expression and chromatin states at T_FH_-related loci.

To validate the interaction between METTL8 and TCF1, coimmunoprecipitation was conducted using HEK293T cells cotransfected with pMigR1-*Mettl8*-*Flag* and pMigR1-*Tcf7* plasmids. The results demonstrated that TCF1 coimmunoprecipitated with METTL8 (Figure 6G). Thus, METTL8 can interact with TCF1 protein, supporting a potential mechanism by which METTL8 influences TCF1-associated chromatin accessibility and 3D genome organization.

To determine whether the phenotypic changes in CD4^+^ T cells are caused by the catalytic activity of METTL8, we used a retroviral transduction system to introduce a point mutation (D220A) at the conserved catalytic residue aspartic acid 220 (27, 28) of METTL8 in Smarta cells. These cells were then adoptively transferred into recipient mice followed by LCMV-Armstrong infection. On day 8 after infection, the D220A mutation abolished the T_FH_-promoting effect of METTL8 overexpression (Figure 6H) and failed to restore TCF1 expression (Figure 6I). These results suggest that catalytic activity of METTL8 is required for the T_FH_-promoting function.

Considering that distinct METTL8 isoforms generated by alternative splicing exhibit differential subcellular localization, residing either in the mitochondria or the nucleolus, we investigated METTL8 distribution in CD4^+^ T cells. Immunofluorescence analysis revealed that METTL8 was present in both the mitochondria and the nucleus (Figure 6J and Supplemental Figure 8C), confirming its dual subcellular localization in this cell type.

### Ginkgolic acid suppresses T_FH_ cell responses through a METTL8-associated mechanism.

A recent study reported that the nuclear localization of METTL8 was regulated by SUMOylation, which could be completely disrupted by ginkgolic acid (GA), a well-known SUMOylation inhibitor (33). To investigate whether GA affects T_FH_ cell differentiation, we treated LCMV-Armstrong–infected *Mettl8*-tdTomato-Flag mice with GA (Figure 7A). The data revealed that METTL8 expression reflected by the MFI of tdTomato was significantly decreased in PD-1^hi^CXCR5^hi^ GC T_FH_ cells upon GA treatment (Figure 7B), suggesting that GA may disrupt METTL8 SUMOylation or METTL8-dependent activity.

To further validate that GA‑mediated suppression of GC T_FH_ cells is associated with METTL8, we treated *Mettl8*^−/−^ Smarta cell–transferred mice (KO mice) with GA following LCMV infection (Figure 7C). In these KO mice, GA treatment did not alter total lymphocyte or Smarta cell numbers (Supplemental Figure 9, A and B). However, in WT recipients, GA led to a significant decrease in PD-1^hi^CXCR5^hi^ and BCL6^+^CXCR5^+^ GC T_FH_ cells (Figure 7, D and E), along with a reduction in PD-1^lo^CXCR5^+^ and SLAM^lo^CXCR5^+^ T_FH_ cells, while the number of CXCR5^−^ non-T_FH_ cells remained unchanged (Supplemental Figure 9, C and D). Similar results were obtained in the direct LCMV infection model (Supplemental Figure 9, E−G). These data indicate that GA inhibits T_FH_ cell differentiation.

Notably, in *Mettl8*‑deficient mice, the frequencies and absolute numbers of PD-1^hi^CXCR5^hi^ and BCL6^+^CXCR5^+^ GC T_FH_ cells were comparable between GA-treated and untreated groups (Figure 7, D and E), indicating that GA exerts little or no additional effect on GC T_FH_ differentiation in the absence of METTL8. Taken together, these data suggest that GA‑mediated suppression of T_FH_ differentiation is associated with METTL8.

Considering that ICI therapy can trigger autoimmune responses and that neoantigen-driven B cell and T_FH_ cell interactions enhance CD8^+^ T cell antitumor immunity (34), we further investigated whether GA treatment could adversely affect ICI therapy. Thus, we administered GA in combination with anti–PD-1 antibody in B16F10 and MC38 subcutaneous tumor models (Figure 7F). The addition of GA did not result in a statistically detectable reduction in the antitumor effect of anti–PD-1 (Figure 7, G and H), suggesting that GA does not compromise the antitumor activity of PD-1 blockade. Furthermore, we tested T_FH_ cell development and function following GA treatment and found that GA impaired T_FH_ differentiation and GC response after anti–PD-1 therapy in both B16F10 (Figure 7I and Supplemental Figure 10) and MC38 (Supplemental Figure 11) tumor models. Therefore, GA treatment inhibits T_FH_ response in anti–PD-1 immunotherapy.

In summary, these results identify METTL8 as a key regulator of T_FH_ differentiation via *Tcf7* mRNA stabilization and 3D genomic remodeling. Furthermore, genetic loss of METTL8 impairs T_FH_ cell development and function. GA treatment suppresses T_FH_ cell responses in a METTL8‑associated manner.

## Discussion

In this study, we identify the m³C methyltransferase METTL8 as a key regulator of T_FH_ cell differentiation in viral infection and tumor immunotherapy models. Using T cell–specific conditional KO mice and adoptive-transfer approaches, we demonstrated that *Mettl8* deficiency impairs T_FH_ differentiation and GC responses in the LCMV infection model and following anti–PD-1 immunotherapy in tumor models. In the infection model, these changes were accompanied by reduced antibody production and increased serum viral burden. Mechanistically, METTL8 exerts its effects through a dual mechanism: it stabilizes *Tcf7* mRNA in an m³C-associated manner to enhance TCF1 expression and cooperates with TCF1-associated chromatin programs at key T_FH_-associated loci. Furthermore, we show that pharmacological treatment with GA suppresses T_FH_ responses without a statistically detectable reduction in anti–PD-1 antitumor activity. Collectively, our findings identify METTL8 as an epitranscriptomic regulator that integrates RNA modification, TCF1-associated transcriptional control, and chromatin architecture to orchestrate T_FH_ cell differentiation.

While METTL8 has been characterized as a mitochondria-localized enzyme that catalyzes m³C modification on mitochondrial tRNAs and regulates mitochondrial translation and respiration (16–18), we observed that METTL8 was localized in both the mitochondria and the nucleus in CD4^+^ T cells. This is consistent with recent reports that alternative splicing generates distinct METTL8 isoforms with differential subcellular localization (35). Notably, our tRNA m³C-seq and Seahorse analyses revealed no significant differences in tRNA m³C modification or mitochondrial respiration between WT and *Mettl8*-deficient T_FH_ cells, suggesting that the nuclear function of METTL8, rather than a bulk mitochondrial metabolic role, may be primarily responsible for regulating T_FH_ differentiation. This finding is particularly striking given that METTL8 has been implicated in m³C regulation across multiple RNA classes, raising the possibility that its functions depend on cell type, transcript context, and subcellular localization.

The dual mechanism suggested by our data, METTL8-associated stabilization of *Tcf7* mRNA and cooperation with TCF1-associated chromatin programs, may represent a coordinated regulatory strategy. This arrangement may help maintain TCF1 at sufficient levels and facilitate engagement of TCF1-associated chromatin programs. Such a dual mechanism may be particularly important for cell fate decisions, where precise and sustained expression of lineage-defining transcription factors is required.

T_FH_ cells play a dual role in human disease: they are essential for protective humoral immunity but also drive autoimmune pathology when dysregulated. Overexpansion of T_FH_ cells leads to excessive GC responses, autoreactive B cell proliferation, and overproduction of high-affinity pathogenic autoantibodies. In the context of cancer immunotherapy, anti–PD-1 treatment expands CXCL13^hi^ GC T_FH_ cells, which can contribute to the development of immune-related adverse events. By identifying METTL8 as a key regulator of T_FH_ differentiation and demonstrating that GA suppresses T_FH_ responses without compromising antitumor immunity, our findings raise the possibility that the METTL8-associated pathway could be explored to limit T_FH_-driven humoral responses during checkpoint blockade while preserving antitumor activity in selected settings. Direct testing in immune-related adverse event models is required.

The reduction of T_FH_ cells in *Mettl8*-deficient mice, without detectable impairment of antitumor immunity in these models, suggests that T_FH_ cells are not strictly required for the measured antitumor response under these conditions. Antitumor immune control in these models may rely substantially on CD8^+^ cytotoxic T lymphocytes, whereas T_FH_-driven humoral responses may play a subordinate role. Thus, the magnitude of T_FH_ reduction observed here may not be sufficient to affect the overall antitumor response. Alternatively, *Mettl8* deficiency may enhance other effector populations, such as T_H_1 cells, that compensate for diminished T_FH_ help, while residual T_FH_ cells may retain a sufficient helper function. Therefore, T_FH_ contribution to antitumor immunity is context dependent and not universally required, warranting further investigation in distinct tumor models.

Several limitations of this study should be acknowledged. First, while we have demonstrated that GA inhibits T_FH_ differentiation in a METTL8-associated manner, GA is a relatively nonspecific SUMOylation inhibitor that may affect other cellular pathways (36). The development of more specific METTL8 inhibitors would be valuable for therapeutic applications. Second, our mechanistic studies were primarily conducted in the LCMV infection model; whether the same mechanisms operate in the context of cancer or autoimmune diseases remains to be fully established. Third, although we observed that *Mettl8* deficiency impaired GC responses and antibody production, the long-term consequences of METTL8 inhibition on humoral immunity and protection against pathogens require further investigation.

GA has been reported to exhibit pleiotropic effects beyond SUMOylation inhibition, including inhibition of fatty acid synthase, nonspecific sirtuin inhibition, and modulation of NF-κB signaling (36). Therefore, while the limited additional effect of GA in *Mettl8*-deficient cells provides genetic evidence that GA suppresses T_FH_ differentiation at least in part through METTL8, we acknowledge that GA may exert additional METTL8-independent effects in WT cells. The development of more selective METTL8 inhibitors is important to further validate the specificity of this regulatory axis.

In summary, our study identifies METTL8 as an important epitranscriptomic regulator of T_FH_ cell differentiation. The findings support a model in which METTL8 stabilizes *Tcf7* mRNA via m³C modification and cooperates with TCF1 protein to organize chromatin architecture at T_FH_-associated loci. This dual mechanism reinforces proper T_FH_ differentiation and GC responses. Genetic or pharmacological perturbation of the METTL8-associated pathway suppresses T_FH_ responses without detectably compromising antitumor immunity in the tested models, suggesting that this pathway may represent a candidate target for selectively modulating T_FH_-driven humoral responses in autoimmune diseases and cancer immunotherapy.

## Methods

Additional methods may be found in the supplemental material.

### Sex as a biological variable.

Both male and female mice were used across the study, although individual experiments were not designed or powered for sex-stratified analysis. Therefore, data were pooled across sexes and no sex-stratified analyses were performed.

### Mice.

*Mettl8*^fl/fl^ and *Mettl8*-tdTomato-Flag mice were generated by Shanghai Model Organisms and Cyagen Biosciences, respectively. C57BL/6, B6.SJL (CD45.1), *Bcl6*^fl/fl^, *Cd4*^cre^, and Smarta mice were from The Jackson Laboratory.

### Human samples.

Skin from patients with untreated lipomas and those with anti–PD-1–treated lung squamous cell carcinoma was obtained from The First Affiliated Hospital of Chongqing Medical University.

### Cell lines.

B16F10, MC38 and HEK293T were purchased from the cell bank of the Chinese Academy of Sciences (Shanghai, China). All cell lines tested negative for mycoplasma contamination. MC38 and HEK293T were cultured in DMEM medium (Hyclone). B16F10 cells were cultured in RPMI-1640 medium (Hyclone). All the media were supplemented with 10% FBS (BBI) and 1% penicillin-streptomycin solution (Gibco).

### Retroviral constructs and transduction.

Mouse *Mettl8* and *Tcf7* cDNAs were cloned into pMigR1-GFP (Addgene, catalog 27490). The D220A point mutation (GAT→GCT) was introduced into mouse *Mettl8* cDNA using primers designed to replace aspartic acid 220 with alanine. Retrovirus was packaged in HEK293T cells as previously described (25). *Mettl8*^–/–^ or littermate control Smarta mice were primed with LCMV-Armstrong (2 × 10^6^ PFU, i.v.). One day later, splenocytes were spin infected with retrovirus at 1,300 g, 37°C for 90 min, cultured with human IL-2 (100 U/mL) and gp61 peptide (250 nM), and spin infection was repeated the next day. For *Tcf7* rescue, 2–5 × 10^5^ retrovirally infected Smarta cells (mixed GFP^+^ and GFP^–^) were transferred into CD45.1 recipients, followed by i.v. infection with 2 × 10^5^ PFU LCMV-Armstrong. GFP^+^ Smarta cells were analyzed at 8 dpi. For cotransfer, 5 × 10^3^ GFP^+^ or GFP^−^ Smarta cells were sorted, mixed 1:1 (1 × 10^4^ total), transferred, and LCMV-Armstrong infected. For D220A mutation, 1 × 10^4^ GFP^+^ Smarta cells were transferred and infected similarly.

### Preparation of single-cell suspensions, antibody staining, and flow cytometry.

Spleens and lymph nodes were crushed through 70 μm cell strainers, and RBCs were lysed. Single-cell suspensions were stained with antibodies as described (37). The fluorochrome-conjugated antibodies were as follows: PE-Cy7 anti-CD19 (1D3, 25-0193-82), PE anti-CD25 (PC61.5, 12-0251-82), PerCP-Cy5.5 anti-CD44 (IM7, 45-0441-82), FITC anti-CD62L (MEL-14, 11-0621-82), APC anti-CXCL13 (DS8CX13, 17-7981-82), APC anti-Fas (15A7, 17-0951-82), PE anti-GL7 (GL7, 12-5902-82), APC anti-ICOS (C398.4A, 17-9949-82), APC anti-IgD (11-26, 17-5993-82), AF660 anti-Ki67 (SolA15, 606-5698-82), APC and PE anti–PD-1 (J43, 17-9985-82 and 12-9985-82), and PE anti–T-bet (4B10, 12-5825-82) from eBiosciences; APC anti-TCF1 (C63D9, 6709S) from Cell Signaling Technology; PE-Cy7 and APC anti-CD45.1 (A20, 110730 and 110714), PE-Cy7 and APC anti-CD45.2 (104, 109830 and 109814), FITC anti-B220 (RA3-6B2, 103206), AF647 anti-BLIMP1 (5E7, 150003), BV421 anti-CXCR5 (L138D7, 145512), and PE anti-SLAM (TC15-12F12.2, 115904) from BioLegend; and BV510 anti-CD4 (RM4-5, 563106), PE anti-CD138 (281-2, 561070), PE anti-BCL6 (K112-91, 561522), and Fixable Viability Stain 780 (565388) from BD Biosciences. For transcription factors, surface-stained cells were fixed and permeabilized with the Foxp3/Transcription Factor Staining Buffer Set (eBiosciences) and then stained. Active caspase-3/7 was detected using the Vybrant FAM caspase-3/7 kit (Invitrogen) according to the manufacturer’s instructions. Data were acquired on a Beckman CytoFLEX and analyzed with FlowJo software V10 (TreeStar).

### ELISA.

Mouse sera were collected at 14 dpi from LCMV-infected *Bcl6*^fl/fl^ *Cd4*^cre^ recipients. LCMV-specific serum IgG was measured by ELISA using an mcKLH-conjugated LCMV peptide as the coating antigen (38). In brief, a Nunc MaxiSorp flat-bottom 96-well plate (eBiosciences) was coated with 1 μg/mL Imject mcKLH-conjugated LCMV-specific peptide (Genscript) overnight and then incubated with serially diluted serum samples. The KLH-specific IgG was detected by HRP-conjugated goat-anti-mouse IgG (H+L) secondary antibody (Thermo Fisher Scientific) coupled with TMB substrate (BD Biosciences). The absorbance at 450 nm was read on the INFINITE M NANO microplate reader (TECAN).

### H&E staining.

For histological analysis, tissues were fixed in 10% neutral buffered formalin for 24–48 h at room temperature, dehydrated through a graded ethanol series, cleared in xylene, and embedded in paraffin wax. Sections were cut at 4 μm thickness using a microtome, mounted on glass slides, and stained with H&E according to standard protocols (39). Images were captured with Pannoramic MIDI (3DHISTECH).

### Immunoblotting.

The protein expression in cells was determined by Western blotting analysis as described (40). Primary antibodies against the following proteins were used: anti-METTL8 (1:1,000, polyclonal, Novus, NBP2-83196) and anti–β-actin (1:1,000, Cell Signaling Technology, 4970). HRP-linked anti-rabbit (1:3,000, Cell Signaling Technology, 7074P2) secondary antibodies were used. The signaling was imaged with a GE ImageQuant LAS 4000.

### m^3^C AlkAniline-seq library construction.

Following the cell sorting strategy of RNA-seq, 1 × 10^7^ T_FH_ cells per sample were prepared. m^3^C AlkAniline-seq was performed by CloudSeq Biotech (Shanghai, China) according to the published procedure with slight modifications (26). Briefly, for mRNA acquisition, RNA samples were subjected to alkaline hydrolysis for fragmentation. For tRNA acquisition, total RNA was size-selected for small RNA fraction (<200 nt) with the MirVana Isolation Kit (Thermo Fisher Scientific). RNA fragments were dephosphorylated with antarctic phosphatase (New England Biolabs) and then incubated in 1 M aniline for cleavage. RNA libraries were constructed with the GenSeq Small RNA Library Prep Kit following the manufacturer’s instructions. Libraries were controlled for quality and quantified using the BioAnalyzer 2100 system (Agilent Technologies). High-throughput sequencing was performed on an Illumina NovaSeq instrument.

### m^3^C AlkAniline-seq data analysis.

Raw data were generated after sequencing, image analysis, base calling, and quality filtering on a sequencer. First, Q30 was used to perform quality control. After adapter-trimming and removing low-quality reads using cutadapt software (v1.9.1), high-quality clean reads were generated. These clean reads were aligned to a reference genome using bowtie2 software (v2.2.4) with end-to-end mode. Raw counts and coverage counts were calculated by bedtools software (v2.24) and in-house scripts and then ratio (defined as: count/coverage) and fold change (defined as: treat-methyl-ratio/input-methyl-ratio) (41) were calculated. Differential m^3^C sites were calculated based on fold change. The sites were annotated with gene information by bedtools software. Heatmaps were generated using the heatmap.2 function from the R package gplots. The input matrix consisted of logCPM values of differentially expressed tRNAs across samples. Hierarchical clustering was performed using Euclidean distance with average linkage clustering. Scatterplots were generated using log_2_‑transformed normalized average expression values from the RNA expression table. RNAs with fold changes above the cutoff (default 2.0) were colored red (upregulated) or green (downregulated); those below the cutoff were colored purple.

### ATAC-seq data analysis.

Bowtie2 with ‘-very-sensitive’ option was used to map the high-quality reads to the mm10 version of the mouse genome. The uniquely properly paired mapped reads were extracted for downstream analysis. MACS2 with the ‘-nomodel’ and ‘-extsize 147’ options was used to call ATAC peaks. DESeq2 was used to identify significantly different (FDR ≤ 0.05) ATAC peaks between different conditions (42).

### Hi-C library construction.

Following the experimental conditions and cell sorting strategy of RNA-seq, 1 × 10^6^ cells were prepared per sample, each in 2 biological replicates. Hi-C was performed using the Hi-C 3.0 protocol as previously described (43). In brief, the sorted cells were cross-linked with 1% formaldehyde for 10 min at 25°C. The cross-linked cells were lysed in 1 mL lysis buffer (10 mM Tris-HCl, pH 8.0, 10 mM NaCl, and 0.2% NP-40) supplemented with protease inhibitor cocktail (MilliporeSigma) at 4°C for 15 min. The nuclei were collected with 900 μL 1× CutSmart buffer (New England Biolabs) and then treated with 50 μL 0.5% SDS at 62°C for 10 min, followed by adding 25 μL 10% Triton X-100 and 145 μL H_2_O to quench SDS. The resulting chromatin was then digested with NUB I (New England Biolabs) restriction enzymes at 37°C overnight. The reaction was stopped by incubation at 65°C for 20 min. The DNA ends were blunted and labeled with biotin by Klenow DNA polymerase in the presence of dCTP, dGTP, dTTP, and biotin-14-dATP, followed by ligation using T4 DNA ligase (New England Biolabs). After reverse cross-linking, DNA was fragmented by sonication. The DNA fragments were then end-repaired, and the biotinylated DNA fragments were captured using Dynabeads MyOne Streptavidin C1 beads (Invitrogen, Thermo Fisher Scientific). The DNA on beads was ligated to the TruSeq adapters and amplified with PCR for library construction. DNA fragments of 200–500 bp were purified from 1% E-gel and sequenced on Illumina HiSeq in paired-read mode with a read length of 150 nt.

### CUT&Tag data analysis.

Cutadapt was used to remove low-quality bases and adapters. Clean reads were mapped to the mm10 genome using bowtie2. MACS2 was used to call CUT&Tag peaks with default settings. The Bam2wig.py script was used to generate bigwig tracks for visualization in IGV (44).

### Statistics.

Data are presented as mean ± SEM. Two‑group comparisons were performed using 2‑tailed unpaired Student’s *t* tests, while multiple‑group comparisons were performed using 1-way ANOVA followed by Tukey’s multiple-comparison test. Tumor growth curves were analyzed by 2-way ANOVA, and survival curves were analyzed by the log-rank test. For figures involving multiple comparisons, the specific statistical test applied is detailed in the respective figure legend. Graphs and statistical computations were generated using GraphPad Prism 8.0.2 (GraphPad Software).

### Study approval.

All animal experiments were reviewed and approved by the Ethics Committee of the University of Science and Technology of China (approval no. USTCACUC192101002). Human skin tissue samples were obtained from patients who provided written informed consent, under protocols approved by the Ethics Committee of The First Affiliated Hospital of Chongqing Medical University (approval no. 2024-581-01).

### Data availability.

The RNA-seq, m^3^C-seq, ATAC-seq, TCF1 and H3K27ac CUT&Tag, and Hi-C data from WT and *Mettl8*^–/–^ T_FH_ cells, along with METTL8-Flag RIP-seq and METTL8 CUT&Tag data from *Mettl8*-tdTomato-Flag T_FH_ cells, have been deposited at the Gene Expression Omnibus under accession number GSE249068. The spatial transcriptome sequencing data have been deposited at the Genome Sequence Archive (GSA) under accession number HRA020801. As GSA‑Human operates under a controlled-access model in accordance with Chinese regulations on human genetic resources and participant privacy protection, these human data are available upon request from the corresponding author. Numerical source data supporting the figures are provided in the Supporting Data Values file.

## Manuscript information

### Author contributions.

JS performed the experiments and analyzed the data with help from JW, XQ, JM, HY, QL, JY, YD, and HM. DC and JL analyzed the RNA-seq, ATAC-seq, TCF1, METTL8 and H3K27ac CUT&Tag, Hi-C, and scRNA-seq data. TH, XH, and XS provided the Hi-C library construction platform and technical support. RY, XZ, and QC provided paraffin-embedded skin sections and spatial transcriptomic profiling of skin tissues. BL and LY provided key technical support and experimental materials. FL, JL, QH, and HW supervised the overall studies and compiled the manuscript. The order of the co-first authors was determined by their relative contributions to the study. JS performed the majority of the experiments, data analysis, and manuscript writing and is therefore listed first. DC analyzed the ATAC-seq, CUT&Tag, Hi-C, and scRNA-seq data and is listed second.

### AI disclosure.

DeepSeek was used for language polishing during the review period from May to June 2026.

### Conflict of interest.

The authors have declared that no conflict of interest exists.

### Funding.

This study was supported by the following:

- The Ministry of Science and Technology of China (2023YFC2306400 to FL).
- University of Science and Technology of China Research Funds of the Double First-Class Initiative (YD9100002508 to FL).
- State Key Laboratory of Immune Response and Immunotherapy 2025 (SE2550000001 to FL).
- Research Funds of Center for Advanced Interdisciplinary Science and Biomedicine of the Institute of Health and Medicine (QYPY20230027 to FL).
- National Natural Science Foundation of China (82370148 to JL and 82322031 to QH).
- R&D Program of Guangzhou National Laboratory (GZNL2023A02011 to JL).
- Natural Science Foundation of Chongqing (CSTB2024NSCQ-JQX0008 to QH).

## Supplementary Material

Supplemental data

Unedited blot and gel images

Supporting data values

## Figures and Tables

**Figure 1:**
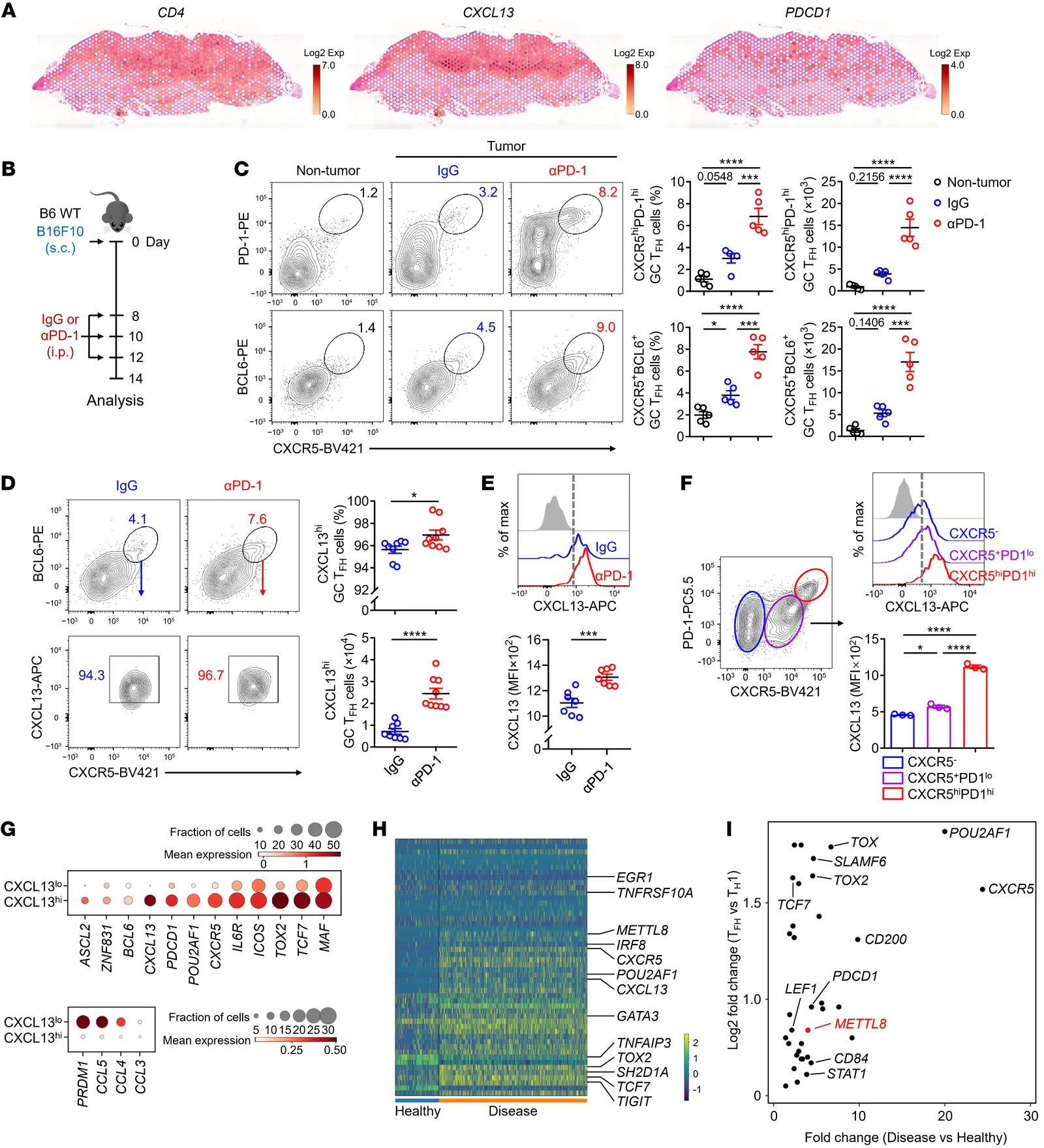
METTL8 is expressed in CXCL13^hi^ CD4^+^ T cells. (**A**) Spatial expression of *CD4*, *CXCL13*, and *PDCD1* in skin lesions from patients with anti–PD-1 treated tumors. (**B**) Schematic diagram of mouse model: B16F10 cells were subcutaneously injected into B6 mice and intraperitoneally injected with anti–PD-1 or IgG every 2 days from days 8 to 12, then analyzed at day 14. (**C**) Representative plots (left) and quantification (right) of PD-1^hi^CXCR5^hi^ and BCL6^+^CXCR5^+^ GC T_FH_ cells (gated on CD44^hi^CD62L^lo^ CD4^+^ T cells) in the TdLNs of non-tumor, IgG‑, or anti–PD‑1–treated tumor mice (*n* = 5/group). (**D**) Frequency and absolute number of CXCL13^hi^ cells (gated on BCL6^+^CXCR5^+^ GC T_FH_ cells) in TdLNs of B16F10 tumor–bearing mice treated with IgG or anti–PD‑1 (*n* = 8–9/group). (**E**) MFI of CXCL13 in BCL6^+^CXCR5^+^ T_FH_ cells in the TdLNs of B16F10 tumor–bearing mice treated with IgG or anti–PD‑1 (*n* = 7/group). (**F**) *Mettl8*-tdTomato-Flag mice were infected with LCMV-Armstrong and analyzed at 8 dpi. Splenic CD44^hi^CD62L^lo^ CD4^+^ cells were gated; CXCL13 expression is shown for PD-1^hi^CXCR5^hi^ GC T_FH_, PD-1^lo^CXCR5^+^ T_FH_, and CXCR5^−^ non-T_FH_ subsets (*n* = 3/group). (**G**) Bubble plot of selected T_FH_/T_H_1-related differentially expressed genes between CXCL13^hi^ and CXCL13^lo^ clusters. (**H**) Heatmap of top 50 differentially expressed genes between CD4^+^ T cells from patients with SS versus healthy controls (scRNA-seq). (**I**) Cross-comparison of gene expression changes in CD4^+^ T cells from patients with disease versus healthy controls and in T_FH_ versus T_H_1 cells. Selected genes shared between the 2 comparisons are highlighted. Mouse data represent 2–3 independent experiments; mean ± SEM. *P* values were calculated using 1-way ANOVA (**C** and **F**) and 2-tailed Student’s *t* test (**D** and **E**). *P < 0.05; ***P < 0.001; ****P < 0.0001.

**Figure 2:**
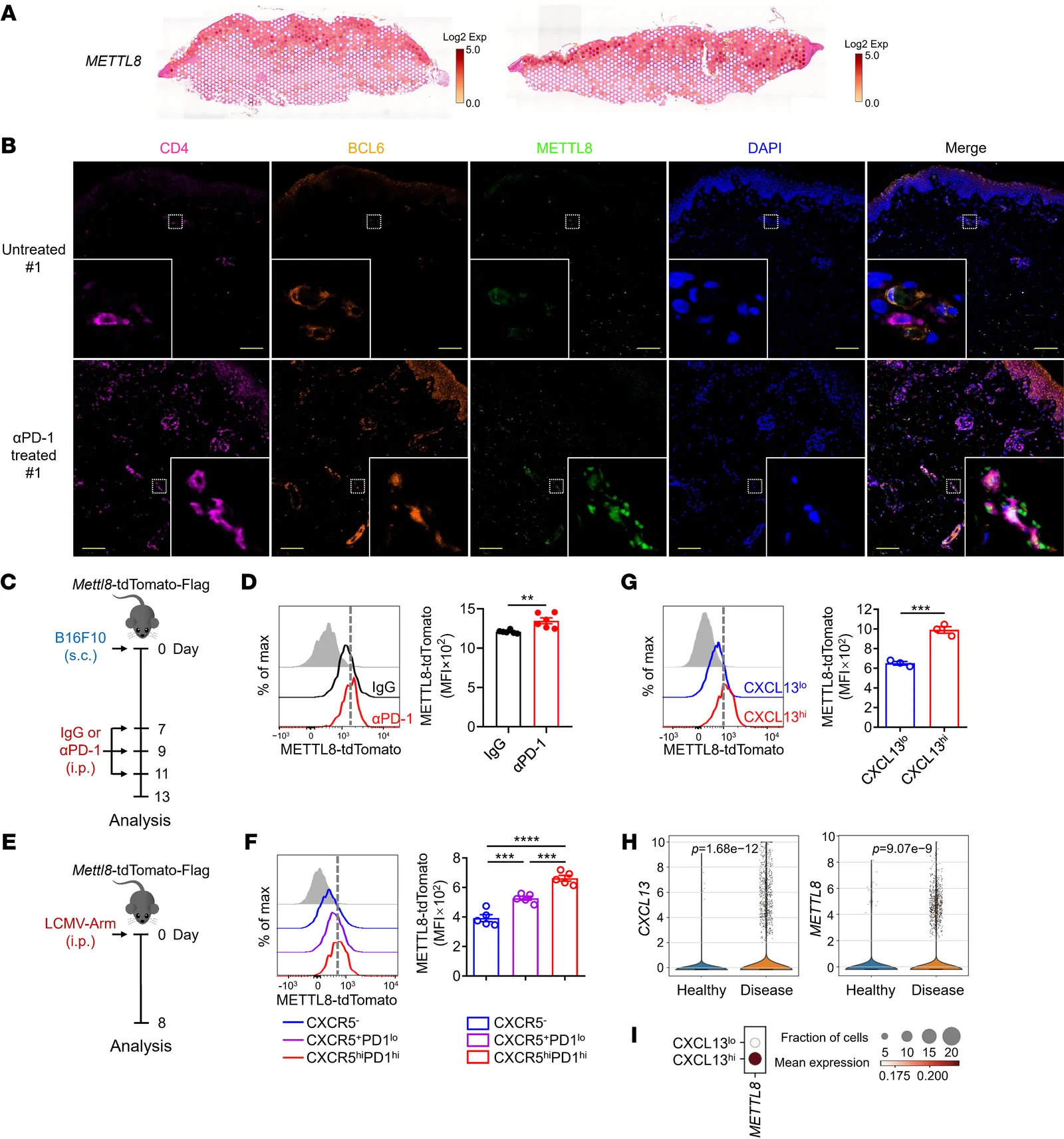
METTL8 is commonly expressed in CXCL13^hi^ CD4^+^ T cells among diverse pathological and experimental settings. (**A**) The distribution and expression of *METTL8* in the skin of patients with anti–PD-1–treated tumors by spatial transcriptome sequencing. (**B**) The expression of *CD4*, *Bcl6*, and *METTL8* in the skin from anti–PD-1–treated or untreated tumor patients by immunofluorescence. Scale bars: 100 μm. (**C**) Schematic of the mouse model: B16F10 cells were subcutaneously injected into *Mettl8*-tdTomato-Flag mice and intraperitoneally injected with anti–PD-1 or IgG every 2 days from days 7 to 11, then analyzed at day 13. (**D**) The MFI of *Mettl8*-tdTomato reporter in PD-1^hi^CXCR5^hi^ GC T_FH_ cells in the spleens of anti–PD-1– or IgG-treated B16F10 tumor–bearing mice (*n* = 6/group). (**E**) Schematic of the mouse model: *Mettl8*-tdTomato-Flag mice were intraperitoneally injected with 2 × 10^5^ PFU LCMV-Armstrong and analyzed at 8 dpi. (**F**) Representative plots and quantification illustrating the expression of *Mettl8*-tdTomato gated on PD-1^hi^CXCR5^hi^ GC T_FH_ cells, PD-1^lo^CXCR5^+^ T_FH_ cells, and CXCR5^−^ non-T_FH_ cells (*n* = 5/group). (**G**) Representative histogram and quantification of *Mettl8*-tdTomato MFI in CXCL13^hi^ and CXCL13^lo^ CD4^+^ T cell subsets (*n* = 3/group). (**H**) Violin plots displaying the expression of *CXCL13* and *METTL8* within healthy controls and patients with SS. (**I**) Bubble plots displaying the expression of *METTL8* within CXCL13^hi^ and CXCL13^lo^ clusters from scRNA-seq analysis. Mouse data represent 2–3 independent experiments; mean ± SEM. *P* values were calculated using a 2-tailed Student’s *t* test. **P < 0.01; ***P < 0.001; ****P < 0.0001.

**Figure 3:**
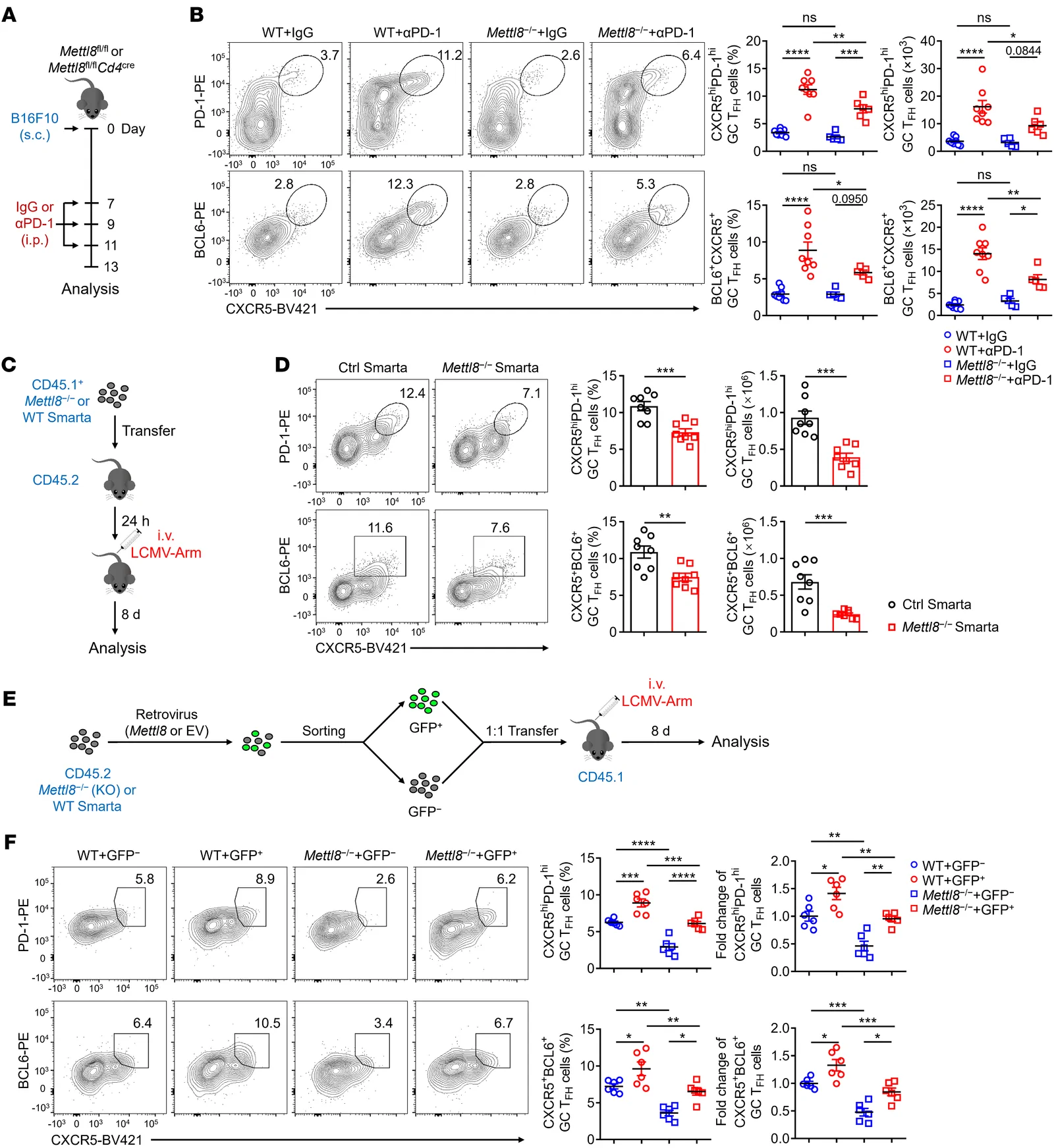
METTL8 promotes T_FH_ cell differentiation. (**A**) Schematic diagram of the mouse model: 2 × 10^5^ B16F10 cells were subcutaneously injected into *Mettl8*^fl/fl^ *Cd4*^cre^ and littermate control mice and intraperitoneally injected with anti–PD-1 or IgG every 2 days from days 7 to 11. Mice were analyzed at day 13. (**B**) Representative plots (left) and quantification (right) of PD-1^hi^CXCR5^hi^ and BCL6^+^CXCR5^+^ GC T_FH_ cells gated on CD44^hi^CD62L^lo^ CD4^+^ T cells in the TdLNs of B16F10 tumor–bearing *Mettl8*^fl/fl^ *Cd4*^cre^ and littermate control mice treated with IgG or anti–PD‑1 antibody (*n* = 5–8/group). (**C**) Schematic of adoptive transfer: CD45.1^+^ *Mettl8*^−/−^ or Ctrl Smarta CD4^+^ T cells were transferred into CD45.2^+^ WT recipients, followed by LCMV-Armstrong (LCMV-Arm) 24 h later, and analyzed at 8 dpi. (**D**) Representative plots (left) and quantification (right) of PD-1^hi^CXCR5^hi^ and BCL6^+^CXCR5^+^ GC T_FH_ cells in CD45.1^+^ Smarta CD4^+^ T cells in spleens of WT recipients (*n* = 8/group). (**E**) Schematic of the cotransfer experiment. CD45.2^+^ *Mettl8*^−/−^ or Ctrl Smarta CD4^+^ T cells were primed in vivo for 24 h and transduced with EV-GFP or *Mettl8*-GFP retrovirus. GFP^+^ and GFP^−^ cells were sorted and cotransferred at a 1:1 ratio into CD45.1^+^ WT recipients, followed by LCMV-Armstrong infection 24 h later and analyzed at 8 dpi. (**F**) Representative plots (left) and quantification (right) of PD-1^hi^CXCR5^hi^ and BCL6^+^CXCR5^+^ GC T_FH_ cells gated on CD45.2^+^ Smarta CD4^+^ T cells in the spleens of mice in **E** (*n* = 6/group). Cell counts were normalized to the corresponding WT GFP^–^ group within each experiment. Data represent 2–3 independent experiments; mean ± SEM. *P* values were calculated using 1-way ANOVA (**B** and **F**) and 2-tailed Student’s *t* test (**D**). *P < 0.05; **P < 0.01; ***P < 0.001; ****P < 0.0001.

**Figure 4:**
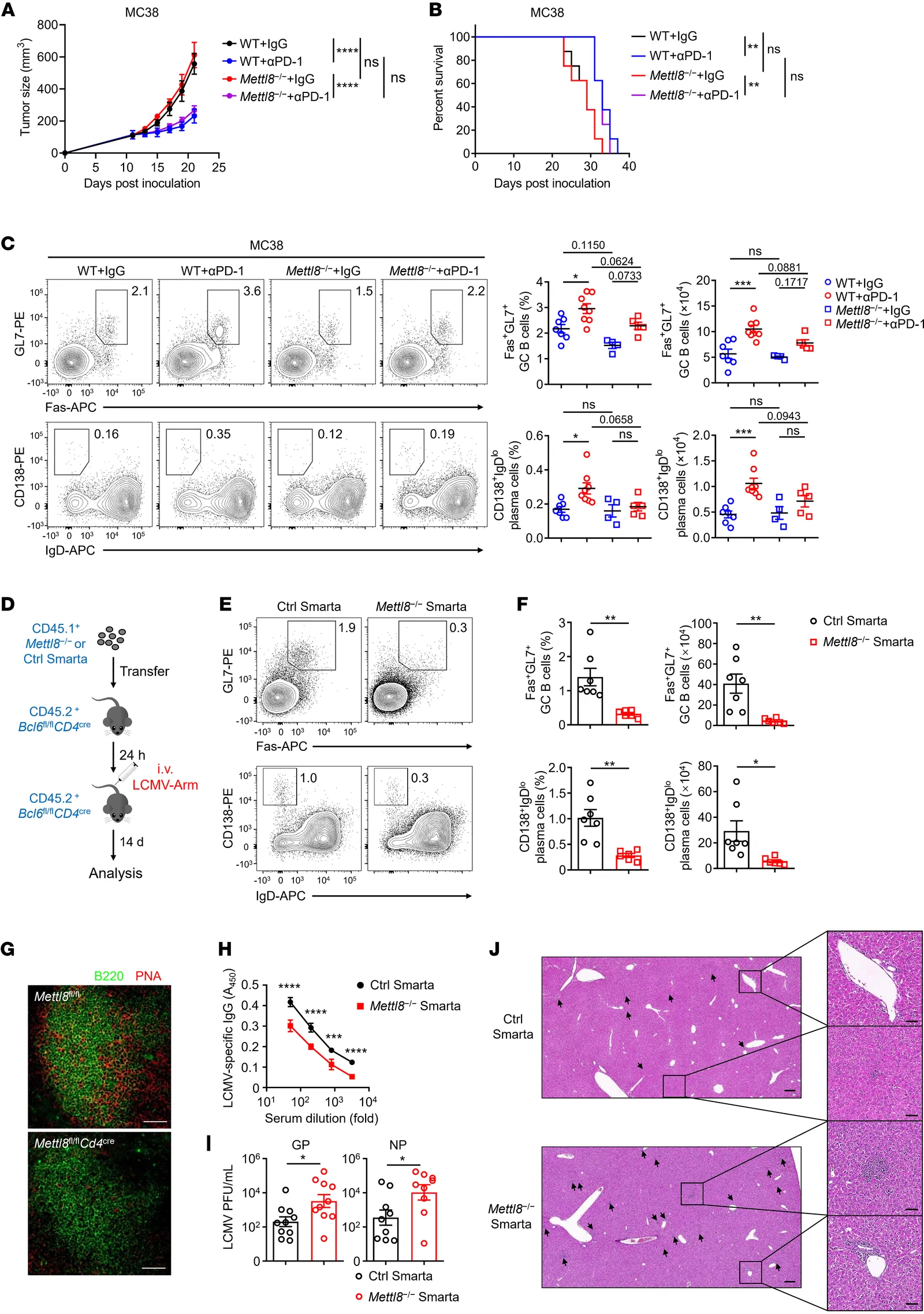
METTL8 promotes T_FH_ cell function. (**A** and **B**) Tumor growth (**A**) and survival (**B**) of MC38-bearing *Mettl8*^fl/fl^ *Cd4*^cre^ and littermate controls, treated with IgG or anti–PD-1 (*n* = 8/group). (**C**) Representative plots (left) and quantification (right) of GL-7^+^Fas^+^ GC B cells and CD138^+^IgD^lo^ plasma cells (gated on B220^+^CD19^+^ cells) in the TdLNs of the above groups (*n* = 4–8/group). (**D**) Schematic of the adoptive transfer model: CD45.1^+^ *Mettl8*^−/−^ or Ctrl Smarta CD4^+^ T cells were transferred into CD45.2^+^ *Bcl6*^fl/fl^ *Cd4*^cre^ recipients, followed by LCMV-Armstrong 24 h later, and analyzed at 14 dpi. (**E** and **F**) Representative plots (**E**) and quantification (**F**) of GC B cells and plasma cells in the spleens of the mice in **D** (*n* = 7–8/group). (**G**) Immunofluorescence of spleens from LCMV-Armstrong–infected *Mettl8*^fl/fl^ *Cd4*^cre^ mice and littermate controls at 8 dpi, indicating B cell follicle (green) and GC (red). Scale bars: 100 μm. (**H**) LCMV-specific IgG in serum of mice in **D** by ELISA (*n* = 5/group). (**I**) Serum samples were collected from *Mettl8*^−/−^ or Ctrl Smarta cell–transferred mice at 4 dpi with LCMV‑Armstrong. Viral titers of the sera were quantified by qPCR using 2 independent primer pairs targeting the LCMV glycoprotein (GP) and nucleoprotein (NP) genes (*n* = 9–10/group). (**J**) Liver samples were collected from *Mettl8*^−/−^ or Ctrl Smarta cell–transferred mice at 8 dpi with LCMV‑Armstrong. H&E staining showing inflammatory cell infiltration (arrows) in indicated groups. Scale bars: 200 μm (left), 50 μm (right). Data represent 2–3 independent experiments; mean ± SEM. *P* values were calculated using 2-way ANOVA (**A**), log-rank test (**B**), 1-way ANOVA (**C**), and 2-tailed Student’s *t* test (**F**, **H**, and **I**), *P < 0.05; **P < 0.01; ***P < 0.001; ****P < 0.0001.

**Figure 5:**
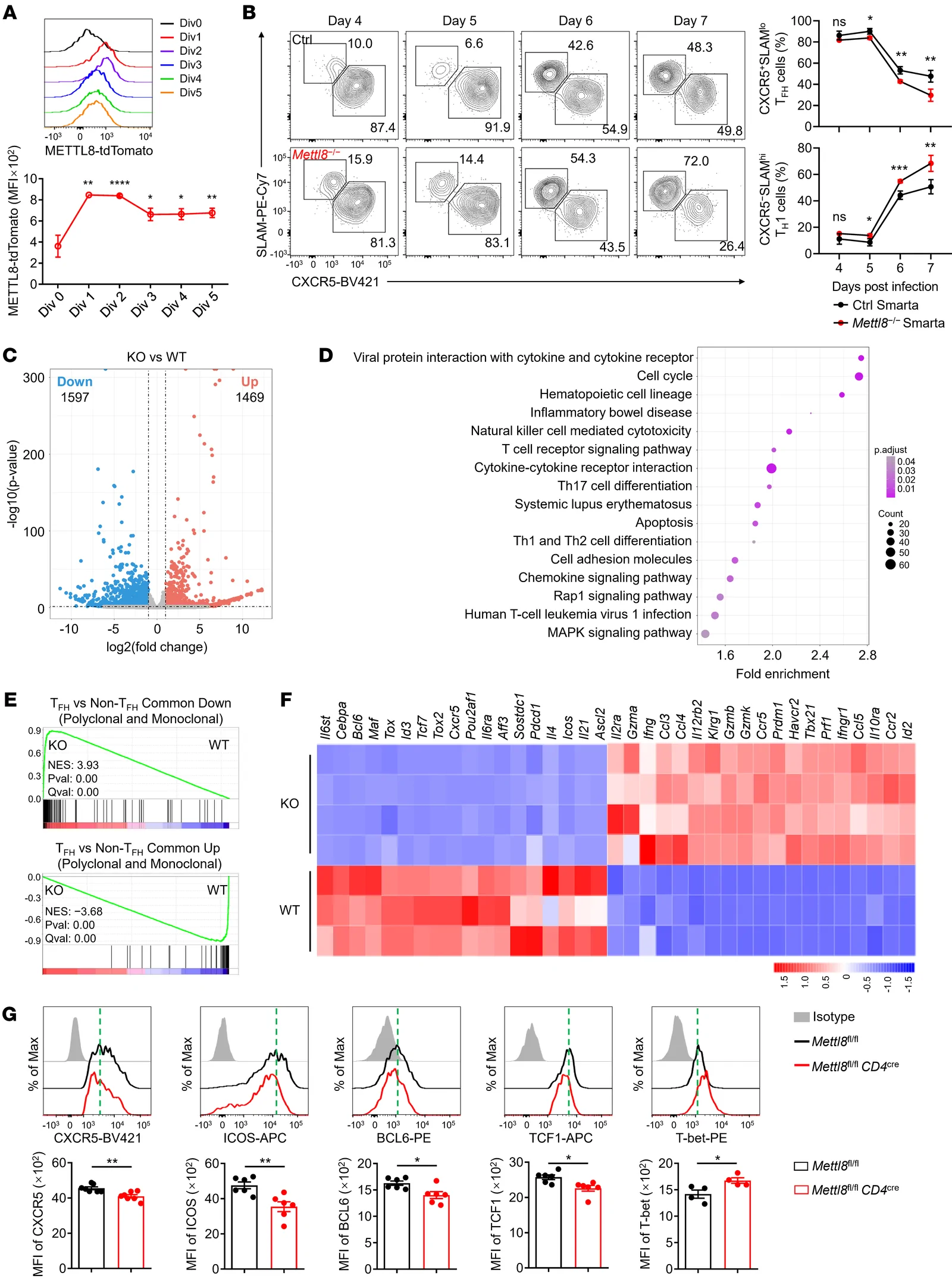
*Mettl8* deficiency impairs T_FH_ differentiation due to altered transcriptional profiles. (**A**) Representative plots (top) and quantification (bottom) of MFI of tdTomato in each division (Div) of CD45.2^+^ Smarta CD4^+^ T cells. All the MFI of tdTomato in divisions 1–5 are compared with that in division 0 (*n* = 4–10/group). (**B**) Representative plots (left) and quantification (right) of the frequency of CXCR5^+^SLAM^lo^ T_FH_ and CXCR5^−^SLAM^hi^ T_H_1 cells in the spleens of *Mettl8*^−/−^ or Ctrl Smarta cell–transferred mice at 4–7 dpi with LCMV‑Armstrong (*n* = 3–4/group). (**C–F**) RNA-seq was conducted on PD-1^hi^CXCR5^hi^ GC T_FH_ cells sorted from spleens of LCMV-Armstrong–infected *Mettl8*^fl/fl^ *Cd4*^cre^ (KO) mice and littermate controls (WT) at 8 dpi. In **C**, Volcano plot illustrates DEGs between GC T_FH_ cells from KO and WT mice. Lines indicate a 2-fold difference between GC T_FH_ cells from KO and WT mice (edgeR, FDR < 0.05). In **D**, KEGG analysis of DEGs obtained from the R package named clusterProfiler. In **E**, GSEA shows the enrichment of T_FH_ versus non-T_FH_ cell-associated downregulated (top) and upregulated (bottom) genes in the transcriptome of KO versus WT GC T_FH_ cells. In **F**, heatmap displaying DEGs related to T_FH_ and T_H_1 cells. (**G**) Representative histograms (top) and cumulative data (bottom) showing the expression of the indicated molecules gated on PD-1^hi^CXCR5^hi^ GC T_FH_ cells (*n* = 4–8/group). For mouse experiments, data represent 2–3 independent experiments; mean ± SEM. *P* values were calculated using a 2-tailed Student’s *t* test. *P < 0.05; **P < 0.01; ***P < 0.001; ****P < 0.0001.

**Figure 6:**
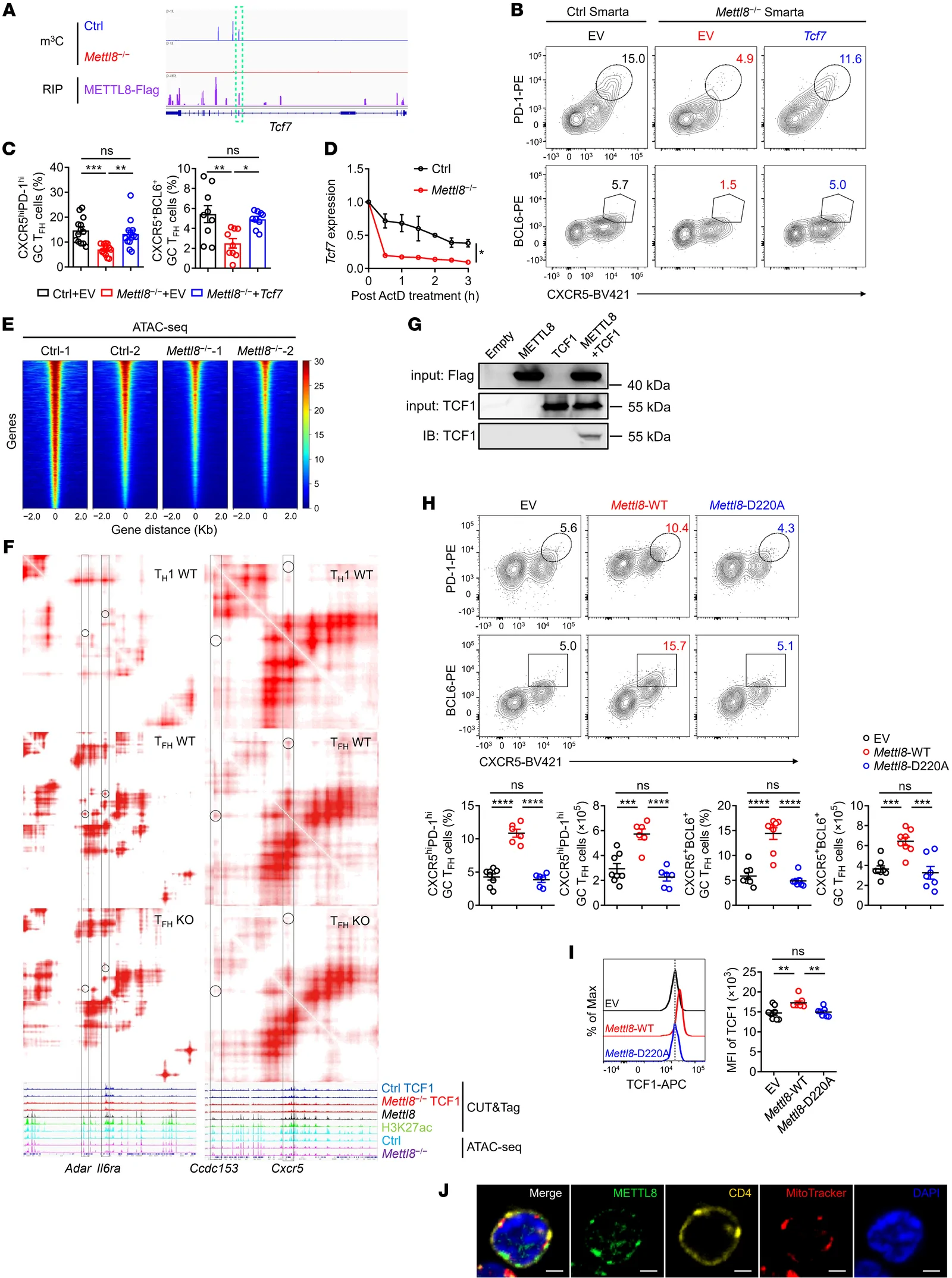
METTL8 promotes m^3^C modification of *Tcf7* mRNA and chromatin accessibility in T_FH_ cells. (**A**) m³C-seq and METTL8-Flag RIP-seq signals mapped to the *Tcf7* transcript. (**B** and **C**) Representative plots (**B**) and quantification (**C**) of PD-1^hi^CXCR5^hi^ and BCL6^+^CXCR5^+^ GC T_FH_ populations gated on Smarta GFP^+^CD4^+^ T cells from the spleens of different recipient mice adoptively transferred with EV or *Tcf7* retrovirus-introduced Smarta cells. Mice were infected with LCMV-Armstrong and analyzed at 8 dpi (*n* = 9–13/group). (**D**) RNA decay assay showing remaining *Tcf7* mRNA detected by RT-qPCR, normalized to *t* = 0 (*n* = 2/group). (**E**) ATAC‑seq heatmaps of local chromatin changes between WT and *Mettl8*^−/−^ PD-1^hi^CXCR5^hi^ GC T_FH_ cells. (**F**) Diamond plots of chromatin interactions in T_H_1, WT, and *Mettl8*^−/−^ (KO) T_FH_ cells at the *Adar*, *Il6ra*, *Ccdc153*, and *Cxcr5* gene loci (top), with CUT&Tag and ATAC-seq tracks and gene structures on the bottom. Vertical lines with circles denote chromatin loop anchors. (**G**) Coimmunoprecipitation of TCF1 with METTL8-Flag in HEK293T cells. (**H**) Representative plots (top) and quantification (bottom) of PD-1^hi^CXCR5^hi^ and BCL6^+^CXCR5^+^ GC T_FH_ cells gated on CD45.2^+^ Smarta CD4^+^ T cells in the spleens of recipient mice transferred with Smarta cells transduced with EV, *Mettl8*‑WT, or *Mettl8*‑D220A retrovirus. Mice were infected with LCMV‑Armstrong and analyzed at 8 dpi (*n* = 6–8/group). (**I**) TCF1 MFI in CXCR5^+^ CD4^+^ T_FH_ cells from the spleens (*n* = 7–8/group). (**J**) Immunofluorescence staining showing METTL8 localization in activated CD4^+^ T cells. Scale bars: 2 μm. Data represent 2–3 independent experiments; mean ± SEM. *P* values were calculated using 1-way ANOVA (**C**, **H**, and **I**) and 2-way ANOVA (**D**). *P < 0.05; **P < 0.01; ***P < 0.001; ****P < 0.0001.

**Figure 7:**
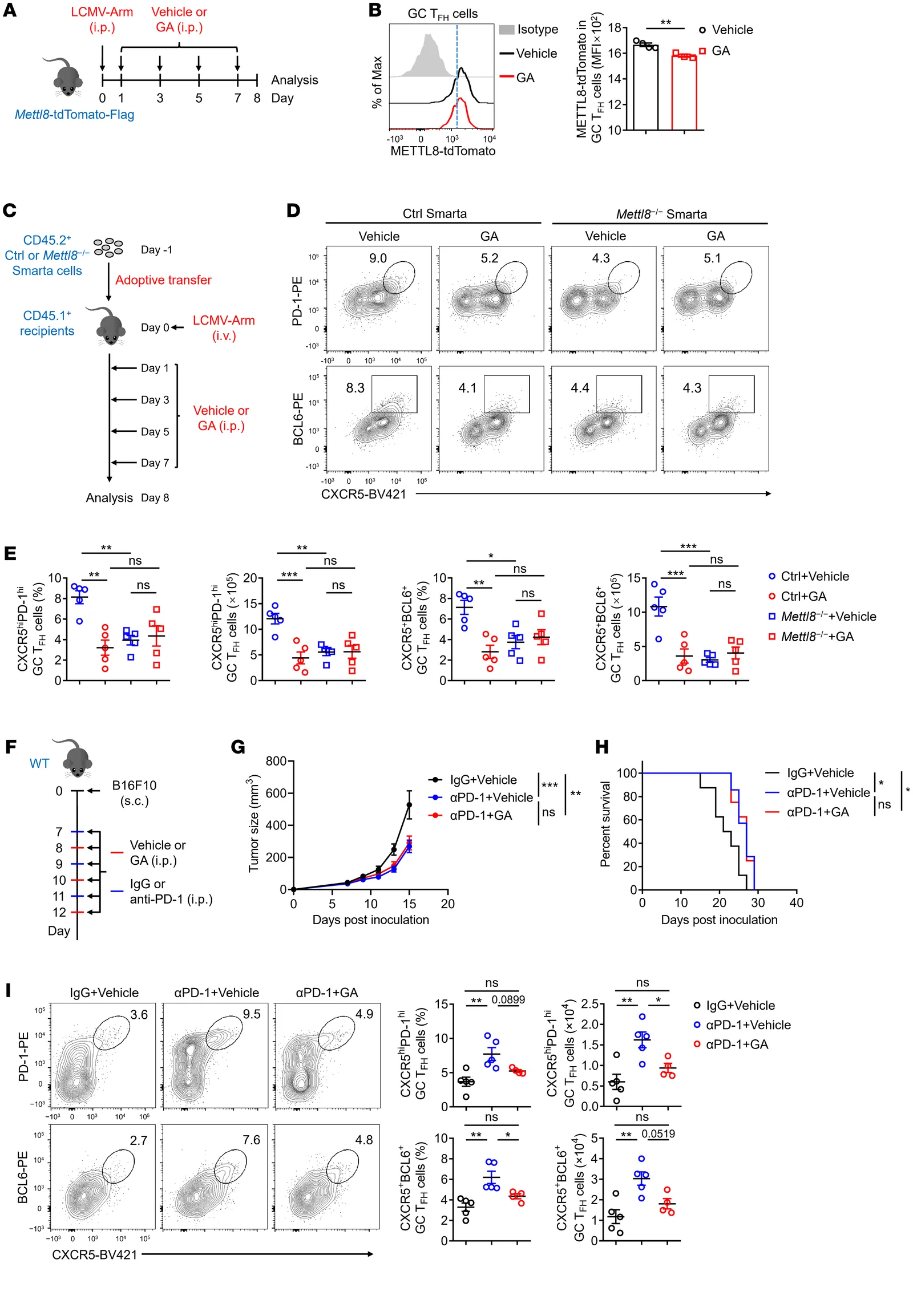
GA treatment reduces T_FH_ cell responses without detectably reducing anti–PD-1 antitumor activity. (**A**) Schematic diagram of GA-treated infection model: *Mettl8*-tdTomato-Flag mice were infected with LCMV-Armstrong on day 0; treated with GA or vehicle on days 1, 3, 5, and 7; and analyzed on day 8. (**B**) Representative histograms and quantification of *Mettl8*-tdTomato reporter MFI in splenic PD-1^hi^CXCR5^hi^ GC T_FH_ cells (*n* = 3–4/group). (**C**) Schematic of the GA-treated adoptive transfer model: CD45.2^+^ Ctrl or *Mettl8*^–/–^ Smarta CD4^+^ T cells were transferred into CD45.1^+^ WT recipients, followed by LCMV-Armstrong 24 h later, and treated with GA or vehicle every 2 days from days 1 to 7 after infection. Spleen cells were analyzed at 8 dpi. (**D** and **E**) Representative plots (**D**) and quantification (**E**) of PD-1^hi^CXCR5^hi^ and BCL6^+^CXCR5^+^ GC T_FH_ cells among donor Smarta cells (*n* = 5/group). (**F**) Schematic of the GA or anti–PD-1 antibody treatment. Mice were subcutaneously injected with B16F10 cells, followed by GA and anti–PD-1 treatment alternately from 7 to 12 days after tumor inoculation. (**G** and **H**) Tumor growth (**G**) and survival curve (**H**) of mice in **F** (*n* = 7–8/group). (**I**) Representative plots (left) and quantification (right) of PD-1^hi^CXCR5^hi^ and BCL6^+^CXCR5^+^ GC T_FH_ cells (gated on CD44^hi^CD62L^lo^ CD4^+^ T cells) in TdLNs of IgG- or anti–PD-1–treated B16F10 tumor–bearing mice, with or without GA (*n* = 4–5/group). Data represent 2–3 independent experiments; mean ± SEM. *P* values were calculated using a 2-tailed Student’s *t* test (**B**), 1-way ANOVA (**E** and **I**), 2-way ANOVA (**G**), and log-rank test (**H**). *P < 0.05; **P < 0.01; ***P < 0.001.

## Version 1 11/16/2026

Electronic publication
