# Supplementary material for: METTL8 orchestrates TFH cell differentiation through m³C-mediated TCF1 regulation in infection and cancer models
Source: J Clin Invest. 2026 Nov 16;136(22):e199747. doi: 10.1172/JCI199747 (PMC13767623; doi:10.1172/JCI199747)
Supplement: Supplementary file 1 [file jci-136-199747-s080.pdf]

Supplemental Methods

Adoptive transfer and LCMV infection

For direct viral infection, *Mettl8*$^{fl/fl}$ *Cd4*^{cre} mice and littermate controls were intraperitoneally (i.p.) infected with 2 × 10^5 plaque-forming units (PFU) of LCMV-Armstrong. For adoptive transfer, naïve Smarta cells were isolated from the LNs of *Mettl8*$^{–/–}$ and littermate control Smarta TCR-transgenic mice. To characterize T_{FH} responses at 8 dpi, 5 × 10^4 Vα2^+ Smarta CD4^+ T cells were intravenously (i.v.) injected into WT or *Bcl6*$^{fl/fl}$ *Cd4*^{cre} recipients, followed by i.v. infection with 2 × 10^5 PFU of LCMV-Armstrong. To assess early division (within 72 h) at T_H1/T_{FH} bifurcation, Smarta cells were labeled with 10 μM Cell Trace Violet (CTV, Invitrogen), and 5 × 10^5 - 1× 10^6 of labeled Vα2^+ Smarta CD4^+ cells were transferred and infected i.v. with 2 × 10^6 PFU LCMV-Armstrong. For basal CTV control, 2 × 10^6 CTV-labeled cells were transferred and left uninfected.

Tumor models

B16F10 cells (2 × 10^5) and MC38 cells (5 × 10^5) were injected subcutaneously into mice. Tumor size was calculated as length × width × width / 2 every 2 days. When the tumor size was larger than 1000 mm^3, the mice were euthanized for ethical consideration.

Anti-PD-1 antibody treatment

For tumor model, anti-PD-1 treatments involved intraperitoneal administration of 200

μg per mouse of anti-PD-1 antibody (clone RMP1-14, Cat. No. A2122, SelleckChem) or rat IgG2a isotype control (clone 2A3, Cat. No. BE0089, Bio X Cell) in 200 μL PBS every two days for a total of three injections, starting when the tumor volume reached 40 mm^3 for B16F10 tumors and 80 mm^3 for MC38 tumors.

Ginkgolic acid treatment

Ginkgolic acid (GA) was purchased from MedChemExpress and suspended in DMSO to 20 mg/mL for storage. For *in vivo* treatment, GA was diluted to 20 μg/mL in a vehicle containing 5% DMSO, 40% PEG300, 5% Tween-80 and 50% PBS. In LCMV infection model, 5×10^4 $V\alpha 2^+$ Smarta cells were transferred into recipients, infected with 2×10^5 PFU LCMV, and GA (200 μg/mouse) was given i.p. every 2 days starting 24 h post-infection; mice were harvested at 8 dpi. For tumor models (B16F10 or MC38), GA was injected i.p. at the same dose every 2 days for 3 doses, starting when tumors reached 40 mm^3 (B16F10) and 80 mm^3 (MC38).

Immunofluorescence staining

For mouse spleen tissue staining, tissue specimens submerged in OCT (Sakura Finetek) compound were quickly frozen in liquid nitrogen and cut into 5 μm thickness. Frozen tissue sections were then fixed with 4% paraformaldehyde for 15 min, and blocked with 1% BSA in PBS for 1 h at room temperature. The tissue sections were then stained with biotinylated PNA (1:20, BA-0074, Vector) and FITC-anti-B220 (1:50, RA3-6B2, 103206, BioLegend) overnight at 4 °C, followed by staining with AF647-labeled anti-

biotin (1:500, 200-602-211, Jackson) for 1 hour at room temperature. For $CD4^+$ T cell staining, cells were sorted from the spleens of LCMV-Armstrong-infected mice at 8 dpi and firstly stained with MitoTracker Red (100 nM, M7512, Invitrogen) for 30 min at 37 °C. Cell smears were prepared by resuspending 1×10^5 cells in PBS, pipetting the suspension onto glass slides, and air-drying to approximately 80% dryness. The smears were then fixed with 4% paraformaldehyde for 15 min, permeabilized with 0.2% Triton X-100 for 10 min, and blocked with 1% BSA in PBS for 1 h at room temperature. Smears were then stained with anti-METTL8 (1:200, A20485, Abclonal), and biotin anti-CD4 (1:50, RM4-5, 100508, BioLegend) overnight at 4 °C, followed by staining with AF488-labeled donkey anti-rabbit IgG (1:1000, A-21206, Invitrogen), and AF647 anti-biotin IgG (1:500, 200-602-211, Jackson) for 1 hour at room temperature. For human skin tissue staining, tissue specimens submerged in paraffin wax and cut into 10 μm thickness. Paraffin-embedded sections were deparaffinized, rehydrated, and subjected to heat-induced antigen retrieval in citrate buffer (pH 6.0) using a microwave. After cooling and washing, sections were permeabilized with 0.2% Triton X-100 for 10 min, and then blocked with 1% BSA and 10% FBS in PBS for 1 h. Sections were stained with anti-METTL8 (1:400, ab122273, Abcam), and anti-BCL6 (1:100, 14-9887-82, Invitrogen) overnight at 4 °C, followed by staining with AF488-labeled donkey anti-rabbit IgG, AF555-labeled donkey anti-mouse IgG (1:500, A-31570 Invitrogen), and AF647-labeled anti-CD4 (1:50, 40568S, Cell Signaling Technology) for 1 hour at room temperature. Then DAPI (Beyotime Biotechnology) was labeled and the slides were washed at least three times with PBS. Coverslips were mounted on slides

using an antifade kit (Beyotime Biotechnology). Confocal images of cryosections were acquired using a Zeiss LSM980 confocal fluorescence microscopy and were processed with Zeiss Zen Microscopy software.

Quantification of LCMV Armstrong viral load in mouse serum by RT-qPCR

Serum viral RNA was extracted from 140 µL of serum using a commercial spin-column RNA isolation kit (QIAGEN). To generate a standard curve, the LCMV Armstrong stock (previously titered by plaque assay) was 10-fold serially diluted in PBS to obtain concentrations from 10^6 to 10^0 PFU/mL. RNA was extracted from each dilution and subjected to RT-qPCR in parallel with serum samples. RT-PCR with SYBR Green (Vazyme) used primers for nucleoprotein (NP, F: GAGGCTTTCTCATCCCAACTAT; R: AAGCTGAAGGCCAAGATCAT) and glycoprotein (GP, F: CATTCACCTGGACTTTGTCAGACTC; R: GCAACTGCTGTGTTCCCGAAAC). Ct values of the serial dilutions were plotted against the $\log_{10}$ of the corresponding titers, and linear regression was used to interpolate viral loads (PFU/mL) in serum samples. Samples with Ct values outside the linear range were reported as below the detection limit. All reactions were run in duplicate, and nuclease-free water and uninfected serum were included as negative controls.

Co-immunoprecipitation (co-IP)

The N-terminally 3×Flag-tagged *Mettl8* (pMigR1) and *Tcf7* plasmids were co-transfected into 293T cells. After 48 h, lysates were immunoprecipitated with

anti-Flag magnetic beads (M2, F1804, Sigma-Aldrich). Magnetic beads were separated from the solution using a magnetic stand. After proper washing, the immunoprecipitated samples were analyzed by immunoblotting with anti-TCF1 (C46C7, 2206, Cell Signaling Technology). Input lysates were probed with anti-TCF1 or anti-Flag to detect input proteins.

RNA decay assay

Mettl8$^{-/-}$ and littermate control Smarta TCR-transgenic mice were infected with 2×10^6 PFU of LCMV-Armstrong. After 24 hours, $CD4^+$ T cells were purified by MACS from the spleens of infected mice. Meanwhile, actinomycin D (MedChemExpress) was added to a final concentration of 10 μM, and cells were harvested at t = 0, 0.5, 1, 1.5, 2, 2.5 and 3 h after actinomycin D treatment. Total RNAs were extracted and subjected to RT-qPCR analysis. Primers used are as follows: *Tcf7*-F (5'−3') CCTGCGGATATAGACAGCACTTC; *Tcf7*-R (5'−3') TGTCCAGGTACACCAGATC CCA.

Metabolic assays

Real-time cell metabolism of T_{FH} cells was measured by using the Seahorse XF Cell Mito stress test kit or a Seahorse XF glycolytic stress test kit (Agilent Technologies) according to the manufacturer's instructions. The T_{FH} cells obtained through flow sorting were placed in the Seahorse assay medium (Agilent Technologies) and supplemented with either 2 mM glutamine (glycolytic stress test) or an addition 10 mM

glucose and 1 mM pyruvate (mito stress test). Each well was inoculated with 25,000 cells per well onto a poly-D-lysine-coated XF 96-well plate (Agilent Technologies), and incubated at 37°C for 1 hour for equilibration. The kit compounds were resuspended in assay medium, and the final concentrations of the compounds in culture wells were 1 μM oligomycin, 1.5 μM FCCP and 0.5 μM rotenone in the mito stress test or 10 mM glucose, 1 μM oligomycin and 50 mM 2-DG in the glycolytic stress test. OCRs and ECARs were measured on a Seahorse XFe 96 analyzer (Agilent Technologies), and data were analyzed using Wave Desktop software (v2.6).

Spatial transcriptome sequencing (Spatial RNA-seq) library construction

Samples were prepared by embedding fresh tissues in OCT (TissueTek Sakura), and stored at -80°C. To assess sample quality, tissue sections were cryosectioned at 10μm by Leica CM1950 cryostat. Then, Hematoxylin & Eosin(H&E) staining, and imaging were performed following 10x Genomics Visium Spatial protocols (CG000160 • Rev C, 10X Genomics). Also, some tissue sections were collected to isolate RNA with miRNeasy Mini Kit (Cat. No. / ID: 217004, QIAGEN). RNA Integrity Number was provided by Agilent 4200 TapeStation, and samples' RIN ≥7 was standard to select samples for further experiment. After quality, cryosection tissue sections at 10μm, and placed the sections within pre-equilibrated Visium Spatial slide. Using Visium Spatial Tissue Optimization Slide & Reagent Kit (PN-1000193, 10X Genomics) and imaging with Leica Aperio CS2 and Leica THUNDER Imager Tissue in order to compare fluorescence images with brightfield images to ensure the appropriate permeabilization

time. According to the appropriate permeabilization time, prepared the libraries by Visium Spatial Gene Expression (PN-1000184, 10X Genomics), imaging with Leica Aperio CS2. Libraries were loaded at 300 pM and sequenced on a NovaSeq 6000 System (Illumina) using a NovaSeq S4 Reagent Kit (200 cycles, 20027466, Illumina). Sequencing was performed using the following read protocol: read 1, 28 cycles; i7 index read, 10 cycles; i5 index read, 10 cycles; and read 2, 90 cycles.

Spatial RNA-seq data analysis

In this study, Space Ranger 1.2.0 was used for data preprocessing, gene expression quantification and point identification. The gene spot matrix was generated by using Visium spatial barcodes, and then point clustering and gene expression analysis were performed. Seurat software 3.2.2 was used to analyse and cluster the spots of samples. Highly variable genes (top 2000) were extracted for principal component analysis (PCA) to reduce the dimensionality of the data and top 30 of significant principal components were used for cluster analysis. Then, using the Uniform Manifold Approximation and Projection (UMAP) and t-Distributed Stochastic Neighbor Embedding (t-SNE) to visualize the clusters. Marker genes for each cluster and subgroup were identified by contrasting gene expression of spots from certain cluster or subgroup to that of others using the Seurat FindMarkers function.

Single cell RNA sequencing (Sc-RNA-seq) data analysis

Highly variable genes (top 1000) were extracted to perform the principal component

analysis (PCA) and top 20 of significant principal components were used for cluster analysis. Clusters were visualized using the Uniform Manifold Approximation and Projection (UMAP). Marker genes for each cluster and subgroup were identified by contrasting gene expression of cells from certain cluster or subgroup to that of others using the Seurat FindMarkers function. The results were visualized using R package.

To enable a systematic analysis of cell–cell communication molecules, cell communication analysis based on the CellPhoneDB, a public repository of ligands, receptors, and their interactions, was applied. Membrane, secreted and peripheral proteins of the cluster of different time point were annotated. Significant mean and cell communication significance (p-value < 0.05) was calculated based on the interaction and the normalized cell matrix achieved by Seurat Normalization. To identify potential interactions between and within fibroblasts and other dermal cell populations, we used CellPhoneDB 2.0 with parameters threshold = 0.25 and iterations = 1000, which contains a curated repository of ligand–receptor interactions and a statistical framework for inferring lineage-specific interactions. Custom R scripts and igraph (V1.2.6) software were used for analyses and to draw the interaction diagrams.

RNA-seq library construction

Mettl8$^{fl/fl}$ *Cd4*^{cre} mice and littermate controls were infected with 2×10^5 PFU LCMV-Armstrong and at 8 dpi, $CD4^+$ $CD44^{hi}$ $CD62L^{lo}$ PD-1^{hi} $CXCR5^{hi}$ T_{FH} cells from the spleens of infected mice were sorted by FACS (BD FACSAria III), each in four biological replicates. RNA was extracted using the Direct-zol RNA MiniPrep kit (Zymo

Research). A total amount of 1 μg RNA per sample was used as input material for the RNA sample preparations. Sequencing libraries were generated using NEBNext UltraTM RNA Library Prep Kit for Illumina (NEB) following manufacturer's recommendations and index codes were added to attribute sequences to each sample. Briefly, mRNA was purified from total RNA using poly-T oligo-attached magnetic beads. Fragmentation was carried out using divalent cations under elevated temperature in NEBNext First Strand Synthesis Reaction Buffer (5X). First strand cDNA was synthesized using random hexamer primer and M-MuLV Reverse Transcriptase (NEB). Second strand cDNA synthesis was subsequently performed using DNA Polymerase I and RNase H. Remaining overhangs were converted into blunt ends via exonuclease/polymerase activities. After adenylation of 3' ends of DNA fragments, NEBNext Adaptor with hairpin loop structure were ligated to prepare for hybridization. In order to select cDNA fragments of preferentially 250-300 bp in length, the library fragments were purified with AMPure XP system (Beckman Coulter). Then 3 μL USER Enzyme (NEB) was used with size-selected, adaptor-ligated cDNA at 37 °C for 15 min followed by 5 min at 95 °C before PCR. Then PCR was performed with Phusion High-Fidelity DNA polymerase, Universal PCR primers and Index (X) Primer. At last, PCR products were purified (AMPure XP system) and library quality was assessed on the Agilent Bioanalyzer 2100 system.

RNA-seq data analysis

STAR (2.7.0a) was used to align RNA-seq raw reads on mm10 genome (UCSC).

Uniquely mapped reads were kept for calculation of gene counts using htseq-count implemented in HTSeq package. Normalization of gene counts and identification of differential expressed genes (FDR < 0.05 and |log2 FC| > 1) were performed by using edgeR.

RIP-seq library construction

Mettl8-tdTomato-Flag mice were infected with 2 × 10^5 LCMV-Armstrong. At day 8 post-infection, $CD4^+$ $CD44^{hi}$ $CD62L^{lo}$ $PD\text{-}1^{hi}$ $CXCR5^{hi}$ T_{FH} cells were sorted from the spleens of infected mice by FACS (BD FACSAria III). RIP-Seq service was provided by Cloud-Seq Biotech (Shanghai, China). The RNA immunoprecipitation assay was carried out with GenSeq RIP kit (GenSeq Inc.) according to the manufacturer's instructions. Briefly, cells were cross-linked with 1% formaldehyde for 10 min at room temperature, quenched with glycine, and lysed in lysis buffer containing protease and RNase inhibitors. Lysates were immunoprecipitated by using anti-Flag antibody (Sigma, clone M2) with PGM beads. After immunoprecipitation, beads were subjected to sequential washes with high-salt buffer (500 mM NaCl) and high-detergent buffer (0.5% SDS and 1% NP-40) to minimize non-specific binding. RNA was eluted in RNase-free water and treated with the GenSeq® rRNA Removal Kit (GenSeq, Inc.) to remove rRNA. Libraries were constructed from the rRNA-depleted RNA using the GenSeq Low Input RNA Library Prep Kit (GenSeq, Inc.) according to the manufacturer's instructions. Library quality and quantity were assessed using the BioAnalyzer 2100 system (Agilent Technologies, Inc.), sequencing was performed on an illumina

NovaSeq instrument with 150bp paired-end reads.

RIP-seq data analysis

Transcriptome high throughput sequencing of RIP-enriched RNA and subsequent bioinformatics analysis were all done by Cloud-Seq Biotech (Shanghai, China). Briefly, paired-end reads were harvested from Illumina NovaSeq 6000 sequencer, and were quality controlled by Q30. After 3' adaptor-trimming and low quality reads removing by cutadapt software (v1.9.3), the high quality reads were aligned to the reference genome with hisat2 software (v2.0.4). Then, HTSeq software (v0.9.1) was used to get the gene level raw count as the expression profiling, and edgeR (v3.16.5) was used to perform normalization and differentially expressed mRNAs were identified by p-value and fold change.

ATAC-seq library construction

Following the experimental condition and cell sorting strategy of RNA-seq, 1×10^5 cells were prepared per sample, each in two biological replicates. Briefly, the sorted cell pellets were washed and treated in lysis buffer for 5 min on ice. The extracted nuclei were re-suspended fragmented buffer including 1.5 μL transposome (novoprotein N248), and incubated at 37 ºC for 30 min. The products were purified with tagment DNA extract beads (novoprotein N245), and then amplified by PCR for 14 cycles with NovoNGS index kit for Illumina (N239). DNA fragments in the range of 200-800 bp were recovered from 2% E-Gel EX Agarose Gels (Invitrogen/ThermoFisher Scientific).

The libraries were sequenced on Illumina HiSeq sequencing systems in paired end 150 bp reads.

Hi-C data analysis

We performed two matched Hi-C comparisons: T_{FH} versus T_H1 and T_{FH} WT versus T_{FH} KO. The input contact maps were provided in .hic format. For downstream HiCExplorer analysis, .hic files were first converted into per-chromosome 10-kb HiCExplorer-compatible .h5 matrices. Briefly, cis-contact records were extracted chromosome by chromosome from the .hic files, binned at 10-kb resolution, and written as sparse HiCExplorer matrices while preserving chromosome bin coordinates. Per-chromosome matrices were then used for TAD and loop calling, and were also merged genome-wide for A/B compartment analysis. All analyses were performed across chr1-19, chrX, and chrY using the same HiCExplorer-based workflow. For A/B compartment analysis, per-chromosome 10-kb matrices were combined into a genome-wide 10-kb matrix and then merged into 100-kb bins with hicMergeMatrixBins. PC1 values were calculated using hicPCA on distance-normalized observed/expected matrices. PC1 vectors were oriented chromosome by chromosome relative to the reference sample in each comparison, and compartment switches were defined as matched 100-kb bins with opposite oriented PC1 signs. TADs and TAD boundaries were called from the 10-kb matrices using hicFindTADs with matched parameters (minDepth 30000, maxDepth 100000, step 10000, minBoundaryDistance 20000, thresholdComparisons 0.05, and delta 0.005). Chromatin loops were identified from the same 10-kb matrices using

hicDetectLoops with matched parameters (maxLoopDistance 2000000, windowSize 10, peakWidth 6, pValuePreselection 0.05, pValue 0.05, peakInteractionsThreshold 5, obsExpThreshold 1.2, and expected mean_nonzero). Shared TAD boundaries were defined by midpoint matching within ±20 kb, and shared loops were defined by matching both loop anchors within ±20 kb. For the T_{FH}-versus-T_H1 comparison, features detected only in T_{FH} or T_H1 were classified as T_{FH}-specific or T_H1-specific, respectively. For the T_{FH} WT-versus- T_{FH} KO comparison, features detected only in WT or KO were classified as WT-specific or KO-specific, respectively. We then cross-compared WT/KO-altered features with the T_{FH}/T_H1 axis to determine whether METTL8-dependent 3D chromatin changes preferentially overlapped T_{FH} -associated or T_H1-associated features.

For diamond plot, raw sequences were aligned to mm10 genome reference and genome contact matrix was generated in 5, 10, 50, 100, 250, and 500 kb resolution used HiC-Pro 3.1.0. Juicer_tools 1.22.01 was applied to convert .pair files to .hic files. Visualization of normalized contact files was performed with Juicebox 2.15. DeepLoop (PMID: 35817982) with pre-trained deep-learning models was applied to enhance loop detection. Specifically, HiCorr was used to perform bias correction, which was further input to pre-trained deep enhance model to detect loops.

CUT&Tag library construction

For CUT&Tag, 1×10^5 cells sorted via RNA-seq (TCF1, H3K27ac) or RIP-seq (METTL8-Flag) strategies were processed using Vazyme kit. Briefly, Cells were sorted

enriched by ConA-magnetic beads and re-suspended in wash Buffer (20 mM HEPES pH 7.5, 150 mM NaCl, 0.5 mM Spermidine; 1 × Protease inhibitor cocktail; 0.05% Digitonin), and then incubated overnight with anti-TCF1 (1:50, C63D9, 2203, Cell Signaling Technology), anti-H3K27ac (1:50, ab4729, Abcam), or anti-METTL8 (1:25, A20485, Abclonal). The next day, beads were washed in Antibody Buffer for twice and incubated with anti-rabbit secondary antibody for 1 hr at a dilution of 1:100. Then the beads were washed for twice and incubated with proteinA/G-Tnp transposome in Chitag. Next, cells were resuspended in 100 μL Tagmentation buffer (10 mM $MgCl_2$ in Chitag Buffer) and incubated at 37 °C for 1 hr. The tagmentation was terminated by adding 1 μL of 10% SDS at 55 °C for 10 min. The DNA fragments added 1 μL spike in (Vazyme) and were extracted by DNA extract beads, then amplified by PCR for 12 cycles with TruePrep index kit for illumine. Each individual library has been paired-end sequenced on an Illumina NovaSeq platform.

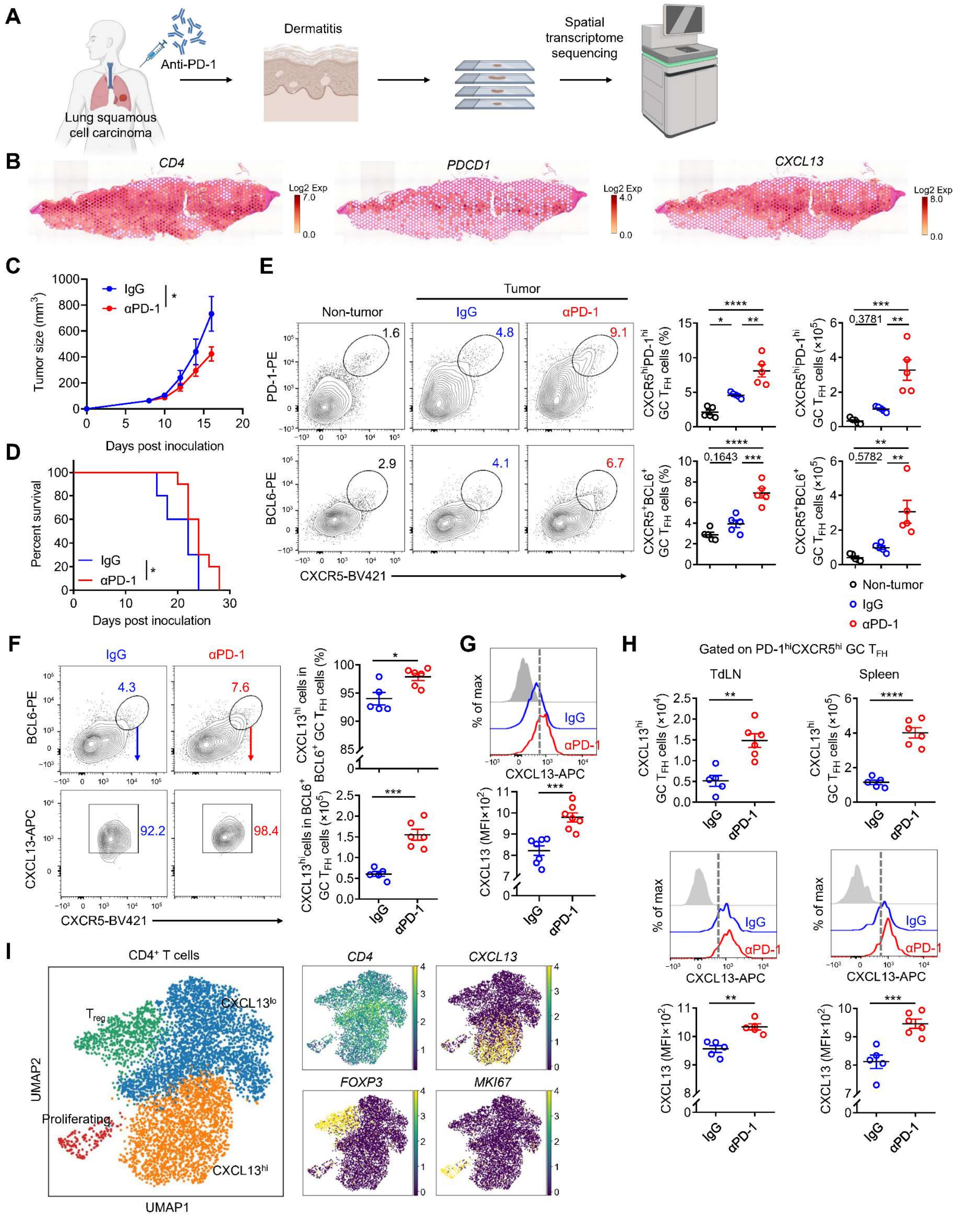

Supplemental Figure 1. CXCL13^{hi} CD4^{+} T cells are commonly detected across diseases and mouse models. (**A**) Schematic diagram of human skin sample collection and spatial transcriptomics: Skin samples from lung squamous cell carcinoma patients who developed autoimmune dermatitis after anti-PD-1 treatment and were analyzed by spatial transcriptome sequencing. (**B**) The distribution and expression of *CD4*, *CXCL13*, *PDCD1* in these skin samples by spatial transcriptome sequencing. (**C and D**) Tumor

growth (C) and survival curve (D) of B16F10 tumor-bearing mice treated with IgG or anti-PD-1 (n = 10/group). (**E**) Representative plots (left) and quantification (right) of PD-1^{hi} CXCR5^{hi} and BCL6^{+} CXCR5^{+} GC T_{FH} cells (gated on CD44^{hi} CD62L^{lo} CD4^{+} T cells) in the spleens of tumor-free and B16F10-bearing mice treated with IgG or anti-PD-1 antibody (n = 5/group). (**F**) Representative plots (left) and quantification (right) of the frequency and absolute number of CXCL13^{hi} cells gated on BCL6^{+} CXCR5^{+} GC T_{FH} cells in the spleens of B16F10-bearing mice treated with IgG or anti-PD-1 antibody (n = 5–6/group). (**G**) Mean fluorescence intensity (MFI) of CXCL13 in BCL6^{+} CXCR5^{+} GC T_{FH} cells in the spleens of B16F10-bearing mice treated with IgG or anti-PD-1 antibody (n = 7/group). (**H**) The absolute number of CXCL13^{hi} cells (top) and the expression of CXCL13 (middle and bottom) gated on PD-1^{hi} CXCR5^{hi} GC T_{FH} cells in the TdLNs (left) and spleens (right) of B16F10-bearing mice treated with IgG or anti-PD-1 antibody (n = 5–6/group). (**I**) Clustering analysis revealing distinct clusters of CD4^{+} T cells in the submandibular glands of SS patients through single-cell RNA sequencing (scRNA-seq). Mouse data represent 2–3 independent experiments, mean ± SEM. P values were calculated using the two-way ANOVA (**C**), Log-rank test (**D**), one-way ANOVA (**E**), and two-tailed Student's *t*-test (**F-H**), $*p < 0.05$; $**p < 0.01$;$***p < 0.001$;$****p < 0.0001$.

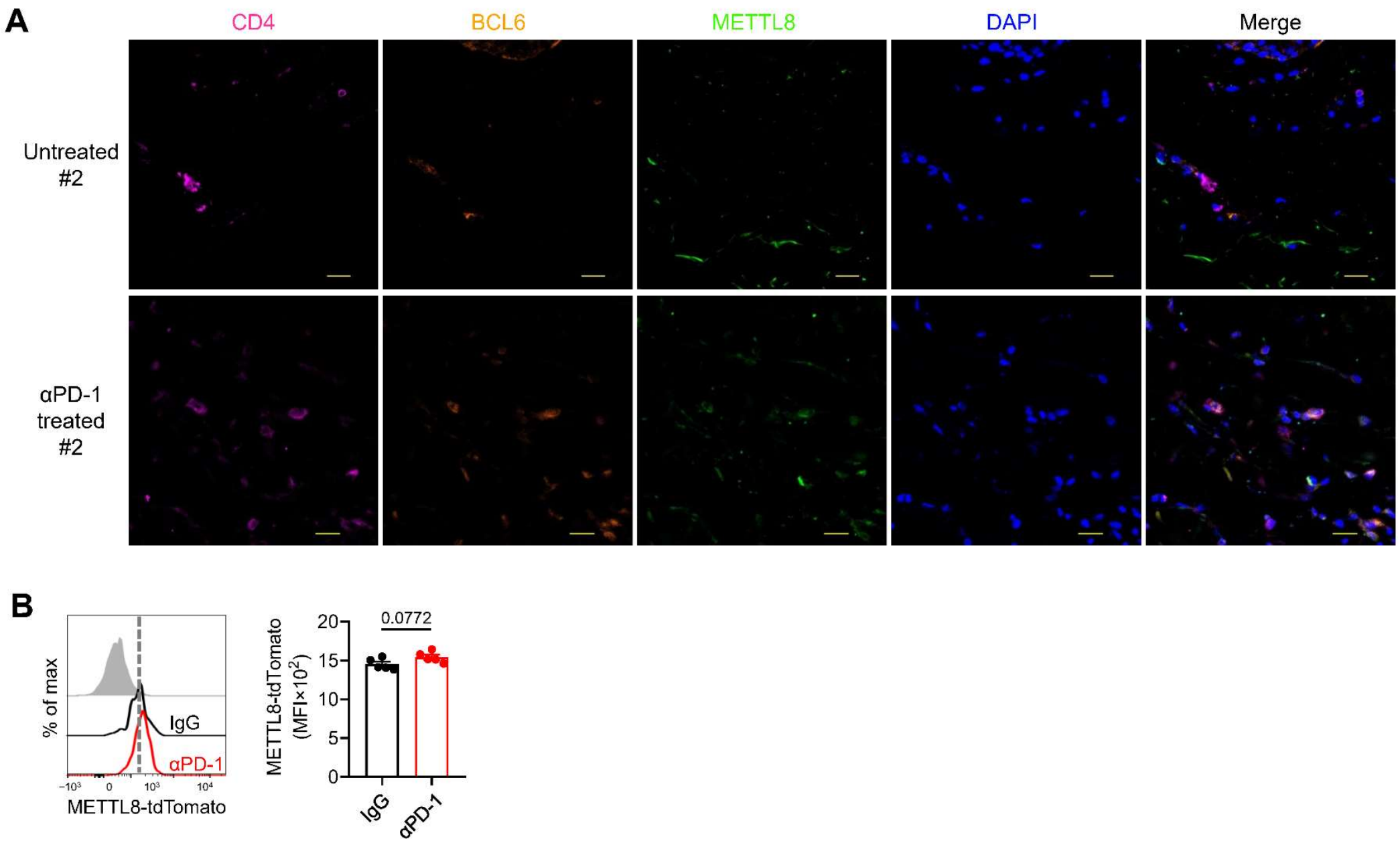

Supplemental Figure 2. The expression of CD4, BCL6 and METTL8 in the skins of anti-PD-1 treated tumor patients. (**A**) The expression of CD4, BCL6, METTL8 and DAPI in the skins from two distinct pairs of anti-PD-1 treated or untreated tumor patients was assessed by immunofluorescence. Scale bar: 20 μm. (**B**) The MFI of METTL8 in CXCR5^{+} CD4^{+} T_{FH} cells in the TdLNs of B16F10-tumor bearing mice treated with IgG or anti-PD-1. Mouse data represent 2–3 independent experiments, n = 5/group, mean ± SEM. P values were calculated using a two-tailed Student's *t*-test.

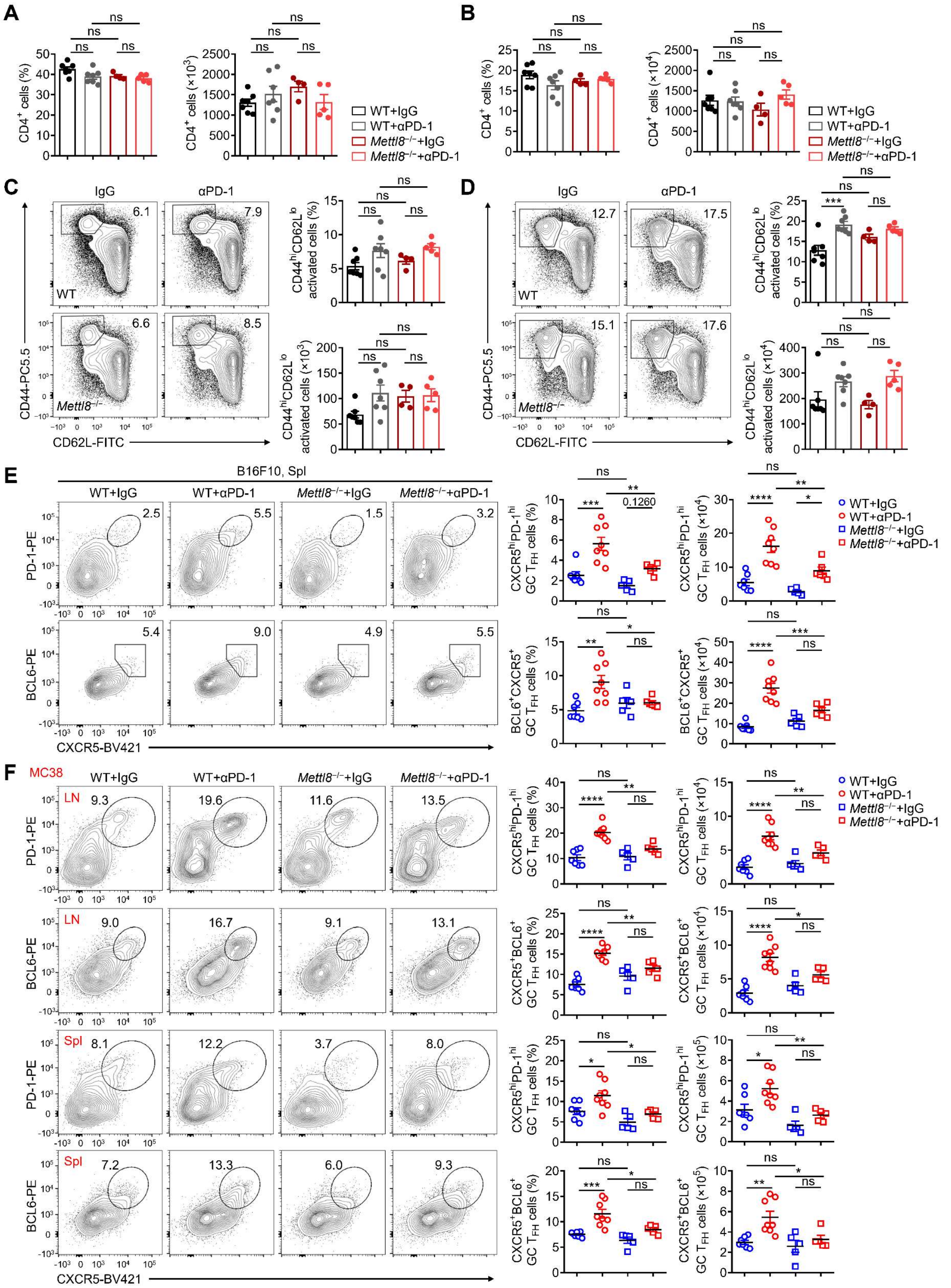

Supplemental Figure 3. METTL8 promotes T_{FH} cell accumulation in anti-PD-1 treated tumor-bearing mice. (**A** and **B**) The proportion and absolute number of $CD4^+$ T cells in the TdLNs (**A**) and spleens (**B**) of B16F10 tumor-bearing *Mettl8*$^{fl/fl}$ *Cd4*^{cre} or littermate control mice treated with IgG or anti-PD-1 antibody (n = 4–7/group). (**C** and

D) Representative plots (top) and cumulative data (bottom) show the proportion and absolute number of CD44^{hi} CD62L^{lo} activated cells gated on CD4^{+} T cells in the TdLNs (**C**) and spleens (**D**) of B16F10 tumor-bearing *Mettl8*$^{fl/fl}$ *Cd4*^{cre} or littermate control mice treated with IgG or anti-PD-1 antibody (n = 4–7/group). (**E**) Representative plots (left) and cumulative data (right) show PD-1^{hi} CXCR5^{hi} and BCL6^{+} CXCR5^{+} GC T_{FH} cells (gated on CD44^{hi} CD62L^{lo} CD4^{+} T cells) in the spleens of B16F10 tumor-bearing *Mettl8*$^{fl/fl}$ *Cd4*^{cre} or littermate control mice treated with IgG or anti-PD-1 antibody (n = 5–8/group). (**F**) Representative plots (left) and cumulative data (right) showing PD-1^{hi} CXCR5^{hi} and BCL6^{+} CXCR5^{+}GC T_{FH} cells (gated on CD44^{hi} CD62L^{lo} CD4^{+} T cells) in the TdLNs (upper) and spleens (lower) of MC38 tumor-bearing *Mettl8*$^{fl/fl}$ *Cd4*^{cre} or littermate control mice treated with IgG or anti-PD-1 antibody (n = 5–8/group). Data represent 2–3 independent experiments, mean ± SEM. P values were calculated using one-way ANOVA, $*p < 0.05$; $**p < 0.01$; $***p < 0.001$; $****p < 0.0001$.

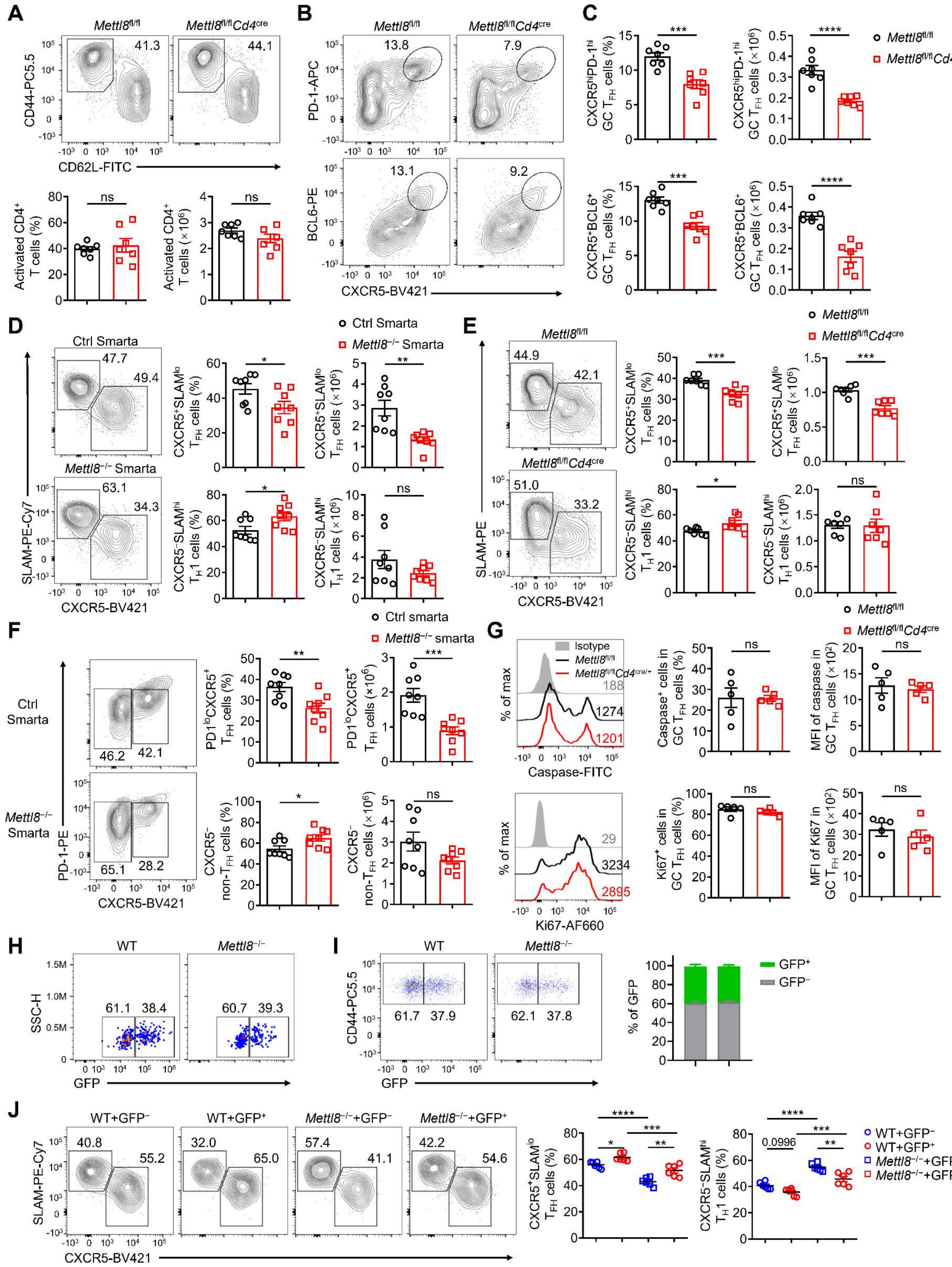

Supplemental Figure 4. Impact of *Mettl8* deficiency on apoptosis and proliferation markers in GC T_{FH} cells. (**A**−**C**) *Mettl8*$^{fl/fl}$ *Cd4*^{cre} mice and littermate controls were infected with LCMV-Armstrong. On 8 dpi, spleens were assessed for CD44^{hi} CD62L^{lo} activated CD4^{+} T cells (**A**, n = 7/group). Subsequently, these cells were analyzed for PD-1^{hi} CXCR5^{hi} and BCL6^{+} CXCR5^{+} GC T_{FH} cells (**B** and **C**, n = 7/group). (**D**) In the

adoptive transfer model, SLAM^{lo} CXCR5^{+} T$_{FH}$ and SLAM^{hi} CXCR5$^{−}$ T$_{H}$1 cells in CD45.1^{+} Smarta CD4^{+} T cells were evaluated in representative plots, proportions, and absolute numbers (n = 8/group). (**E**) Similarly, in the directly infected model, SLAM^{lo} CXCR5^{+} T$_{FH}$ and SLAM^{hi} CXCR5$^{−}$ T$_{H}$1 cells in activated CD4^{+} T cells from *Mettl8*$^{fl/fl}$ *Cd4*^{cre} mice and littermate controls were evaluated in representative plots, proportions, and absolute numbers (n = 7/group). (**F**) Representative plots (left) and cumulative data (right) showing the frequencies and absolute numbers of PD-1^{lo} CXCR5^{+} T$_{FH}$ and CXCR5$^{−}$ non-T$_{FH}$ cells in CD45.1^{+} Smarta CD4^{+} T cells (n = 8/group). (**G**) Expression of caspase and Ki67 in PD-1^{hi} CXCR5^{+} GC T$_{FH}$ cells was depicted in representative plots, and proportions and MFI of these cells were quantified (n = 5/group). (**H**) Ratio of GFP^{+} to GFP$^{−}$ Smarta cells before transfer in the co-transfer experiment. (**I**) Representative plots (left) and cumulative data (right) showing the ratio of GFP^{+} to GFP$^{−}$ Smarta cells at 8 dpi (n = 6/group). (**J**) In the co-transfer experiment, representative plots and proportions of CXCR5^{+} SLAM^{lo} T$_{FH}$ and CXCR5$^{−}$ SLAM^{hi} T$_{H}$1 cells were presented (n = 6/group). Data represent 2–3 independent experiments, mean ± SEM. P values were calculated using two-tailed Student's *t*-test (**A**-**G**), and one-way ANOVA (**J**), *$p < 0.05$; **$p < 0.01$; ***$p < 0.001$; ****$p < 0.0001$.

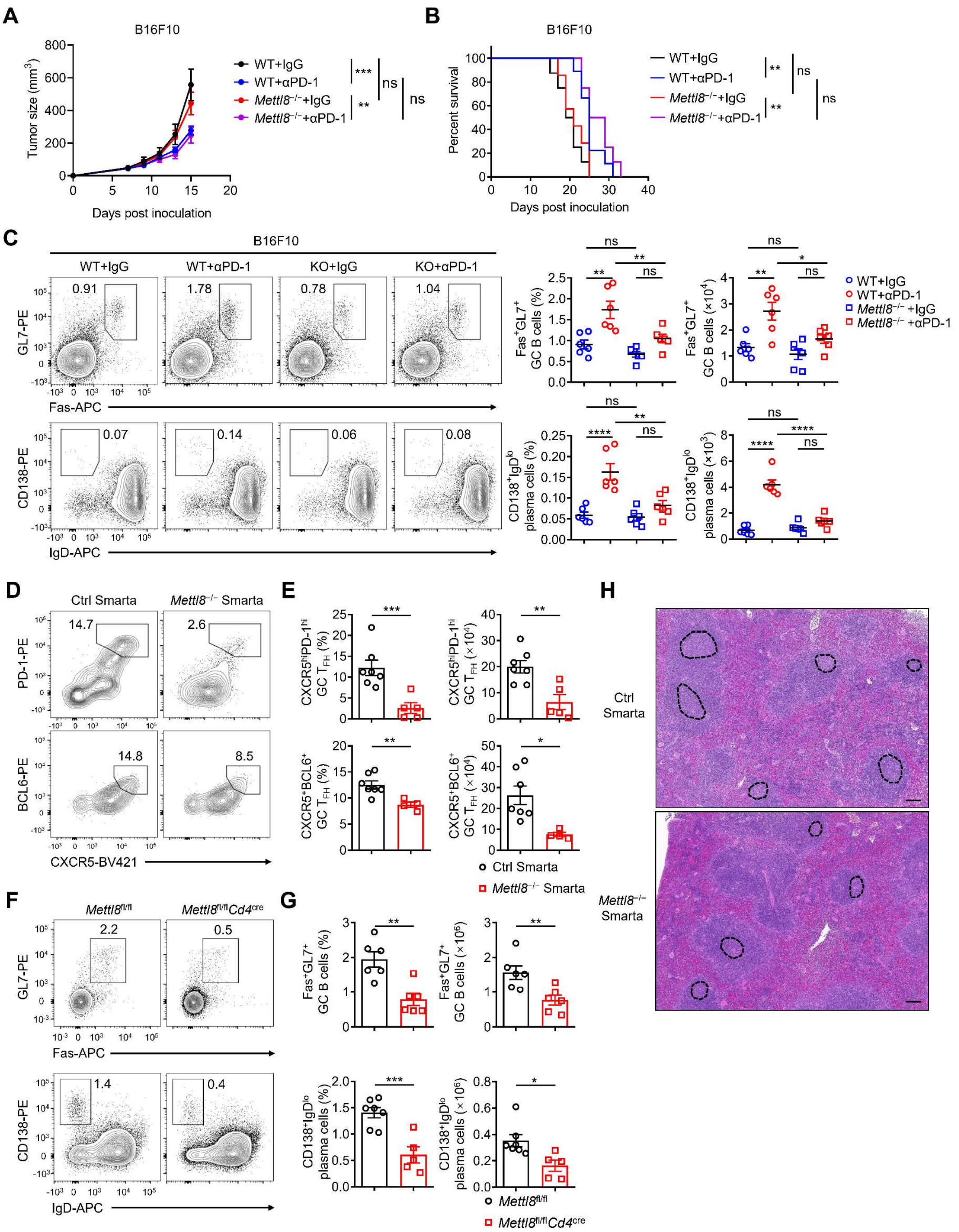

Supplemental Figure 5. Impact of impaired GC T_{FH} differentiation on B cell responses. (**A** and **B**) Tumor growth (**A**) and survival curve (**B**) of B16F10 tumor-bearing *Mettl8*$^{fl/fl}$ *Cd4*^{cre} or littermate control mice treated with IgG or anti-PD-1 (n = 7–9/group). (**C**) Representative plots and statistical analysis showing GL-7^{+} Fas^{+} GC B cells and CD138^{+} IgD^{lo} plasma cells (gated on B220^{+} CD19^{+} cells) in the TdLNs of B16F10 tumor-bearing *Mettl8*$^{fl/fl}$ *Cd4*^{cre} or littermate control mice treated with IgG or

anti-PD-1 (n = 6/group). (**D** and **E**) In *Bcl6*$^{fl/fl}$ *Cd4*^{cre} recipients of the adoptive transfer model, PD-1^{hi} CXCR5^{hi} and BCL6^{+} CXCR5^{+} GC T_{FH} cells in CD45.1^{+} Smarta CD4^{+}T cells were assessed in spleens, and frequency (**D**) and absolute number (**E**) of these cells were determined (n = 4–7/group). (**F** and **G**) *Mettl8*$^{fl/fl}$ *Cd4*^{cre} mice and littermate controls were infected with LCMV-Armstrong. On 8 dpi, GL-7^{+} Fas^{+} GC B cells and CD138^{+} IgD^{lo} plasma cells were detected in the spleen. Frequency (**F**) and absolute number (**G**) of these cells were quantified (n = 5–7/group). (**H**) Spleen samples were collected from *Mettl8*$^{-/-}$ or Ctrl Smarta cell-transferred mice at 8 dpi with LCMV-Armstrong. H&E staining showing follicle-associated pale-staining regions morphologically consistent with germinal centers (shown by circles) in the spleens of the indicated groups. Scale bar: 100 μm. Data represent 2–3 independent experiments, mean ± SEM. P values were calculated using the two-way ANOVA (**A**), Log-rank test (**B**), one-way ANOVA (**C**), and two-tailed Student's *t*-test (**E** and **G**), *$p < 0.05$; **$p < 0.01$; ***$p < 0.001$; ****$p < 0.0001$.

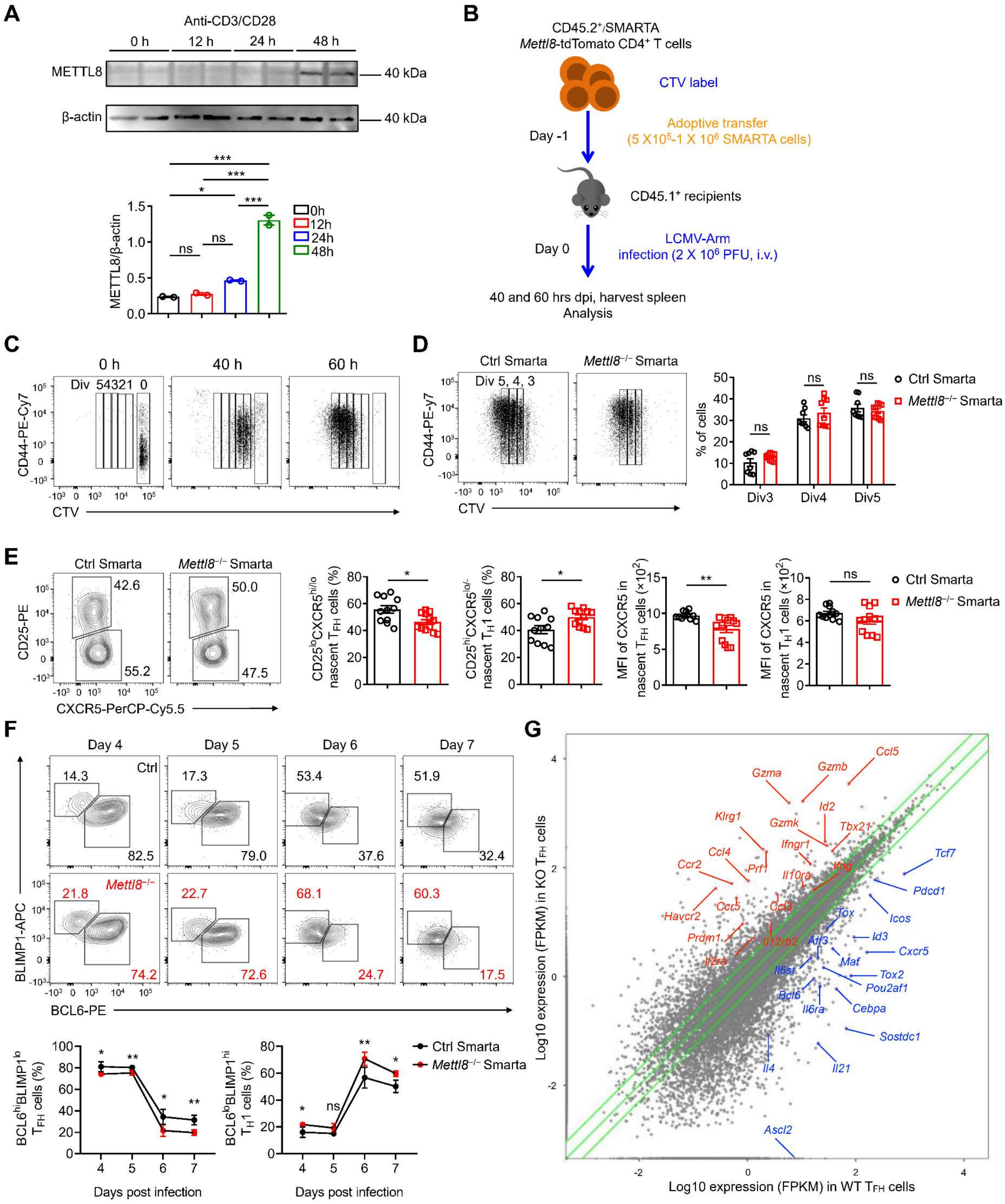

Supplemental Figure 6. Impact of *Mettl8* deficiency on TFH and TH1 cell differentiation. (**A**) METTL8 expression in αCD3/CD28-stimulated CD4$^+$ T cells was determined by western blotting, with the ratio of METTL8 to β-actin expression presented (n = 2/group). (**B**) CD4$^+$ T cell activation and early division were schematically diagrammed. *Mettl8*-tdTomato Smarta CD4$^+$ T cells were labeled with CTV and adoptively transferred at 0.5-1 × 10^6 cells/recipient, followed by intravenous

infection with 2 × 10^6 PFU LCMV-Armstrong. (**C**) $CD45.2^+$ Smarta $CD4^+$ T cells in recipient spleens were identified and assessed for CD44 expression (an activation marker) and CTV dilution 40 h and 60 h later. (**D**) In the $CD4^+$ T cell activation and early division model, WT or *Mettl8*$^{-/-}$ Smarta $CD4^+$ T cells were labeled with CTV, and on 60 hours post-infection, the frequency of cells in third to fifth divisions was assessed in representative plots (n = 7–9/group). (**E**) Representative plots (left) and cumulative data (right) display the frequency of $CD25^{lo}$ $CXCR5^{hi/lo}$ nascent T_{FH} cells and $CD25^{hi}$ $CXCR5^{lo/-}$ nascent T_H1 cells, and the MFI of CXCR5 in these two subsets in division five (n = 11–12/group). (**F**) Representative plots (top) and statistical analysis (bottom) displaying the frequency of $BCL6^{hi}$ $Blimp1^{lo}$ T_{FH} and $BCL6^{lo}$ $Blimp1^{hi}$ T_H1 cells in the spleens of *Mettl8*$^{-/-}$ or Ctrl Smarta cell-transferred mice at 4-7 dpi with LCMV-Armstrong (n = 4/group). (**G**) Scatter-plots showing DEGs between WT and *Mettl8*$^{-/-}$ (KO) T_{FH} cells. For mouse experiments, data represent 2–3 independent experiments, mean ± SEM. P values were calculated using a two-tailed Student's *t*-test, $*p < 0.05$; $**p < 0.01$; $***p < 0.001$.

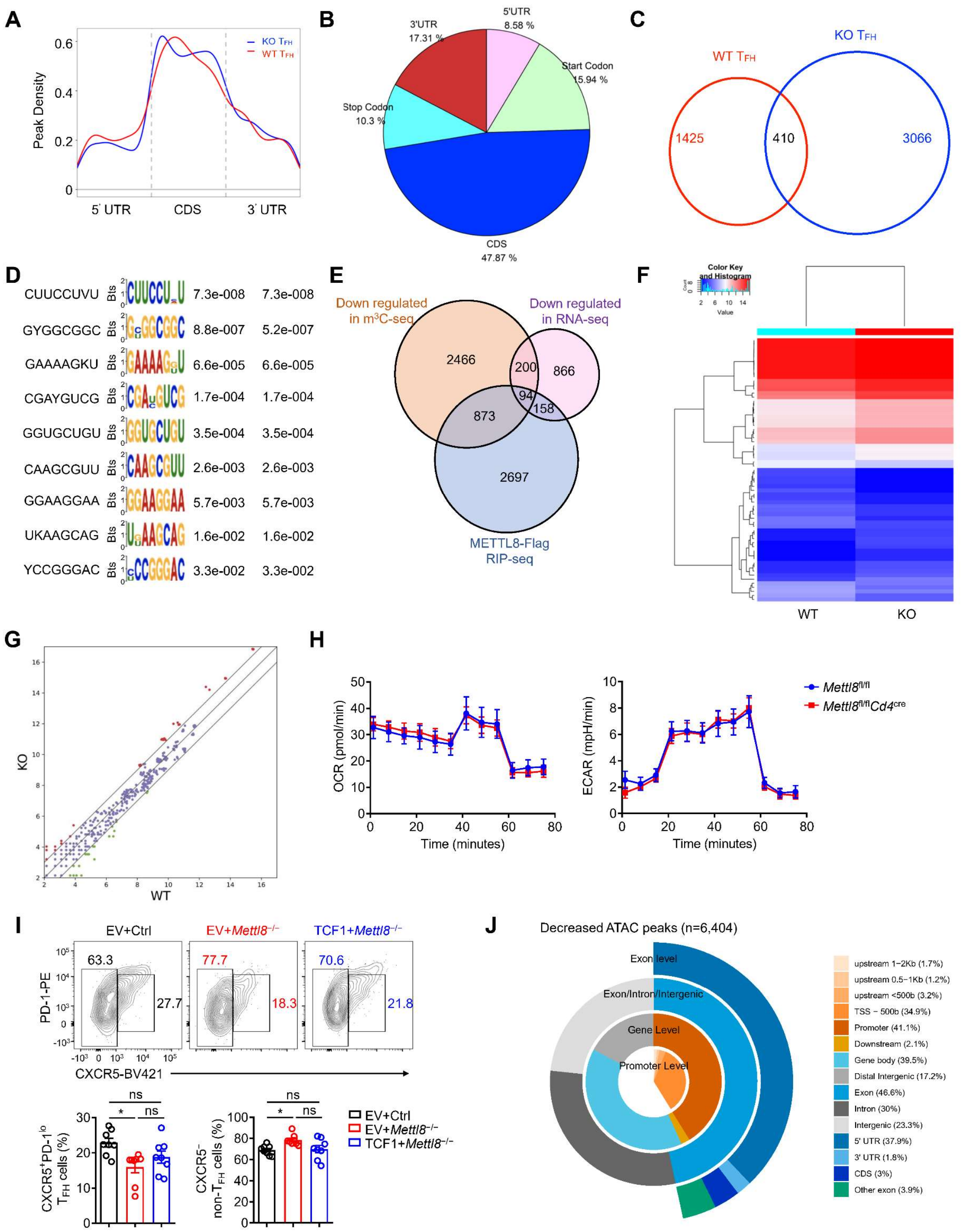

Supplemental Figure 7. *Mettl8* deficiency alters m^{3}C modification in T$_{FH}$ cells. (**A**) Global depiction of m^{3}C sites on mRNA in WT and *Mettl8*$^{-/-}$ (KO) T$_{FH}$ cells. (**B**) Pie chart illustrating the distribution of m^{3}C sites on mRNA. (**C**) Venn plot displaying the genes containing differential m^{3}C sites between WT and *Mettl8*$^{-/-}$ (KO) T$_{FH}$ cells. (**D**) Identification of sequence motifs within m^{3}C peaks. (**E**) The Venn plot illustrates the overlap of down-regulated genes from RNA-seq, m^{3}C-seq, and METTL8-binding

genes from RIP-seq. (**F** and **G**) Heatmap (**F**) and scatter plot (**G**) showing differentially detected tRNA sites identified by tRNA m^3C-seq. (**H**) OCR (left) and ECAR (right) were measured in WT and *Mettl8*$^{-/-}$ T_{FH} cells isolated from LCMV-infected mice at 8 dpi (n = 5–6/group). (**I**) In the transduction experiment, representative plots and proportions of PD-1^{lo} $CXCR5^{+}$ T_{FH} and $CXCR5^{-}$ non-T_{FH} cells were presented (n = 8/group). (**J**) A pie chart illustrating the enriched region of decreased ATAC peaks. For mouse experiments, data represent 2–3 independent experiments, mean ± SEM. P values were calculated using one-way ANOVA, $*p < 0.05$.

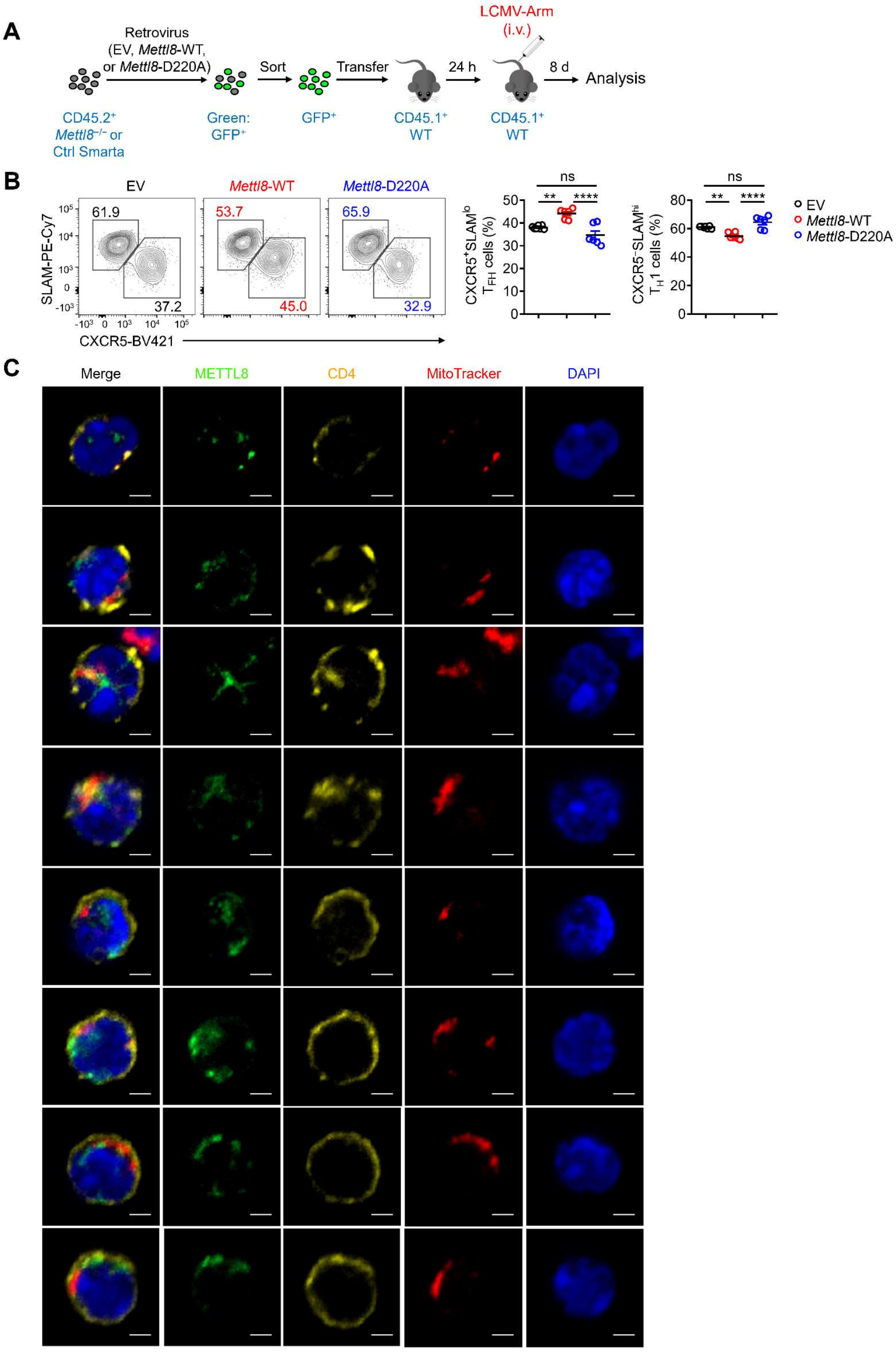

Supplemental Figure 8. METTL8 is present in both mitochondria and nuclei in CD4$^+$ T cells. (**A**) Schematic diagram of the D220A mutation experiment. CD45.2$^+$ *Mettl8*$^{-/-}$ or Ctrl Smarta CD4$^+$ T cells were primed *in vivo* for 24 hours and transduced with EV, *Mettl8*-WT or *Mettl8*-D220A retrovirus. GFP$^+$ cells were sorted and transferred into CD45.1$^+$ WT recipients, followed by LCMV-Armstrong infection 24 h

later and analyzed at 8 dpi. (**B**) Representative plots and statistic analysis showing $CXCR5^{+}$ $SLAM^{lo}$ T_{FH} and $CXCR5^{-}$ $SLAM^{hi}$ $T_{H}1$ cells gated on $CD45.2^{+}$ Smarta $CD4^{+}$ T cells in the spleens of mice in **A** (n = 6–7/group). (**C**) Immunofluorescence staining showing the subcellular localization of METTL8 in activated $CD4^{+}$ T cells. Scale bar: 2 μm. Data represent 2–3 independent experiments, mean ± SEM. P values were calculated using one-way ANOVA, $^{**}p < 0.01$; $^{***}p < 0.001$; $^{****}p < 0.0001$.

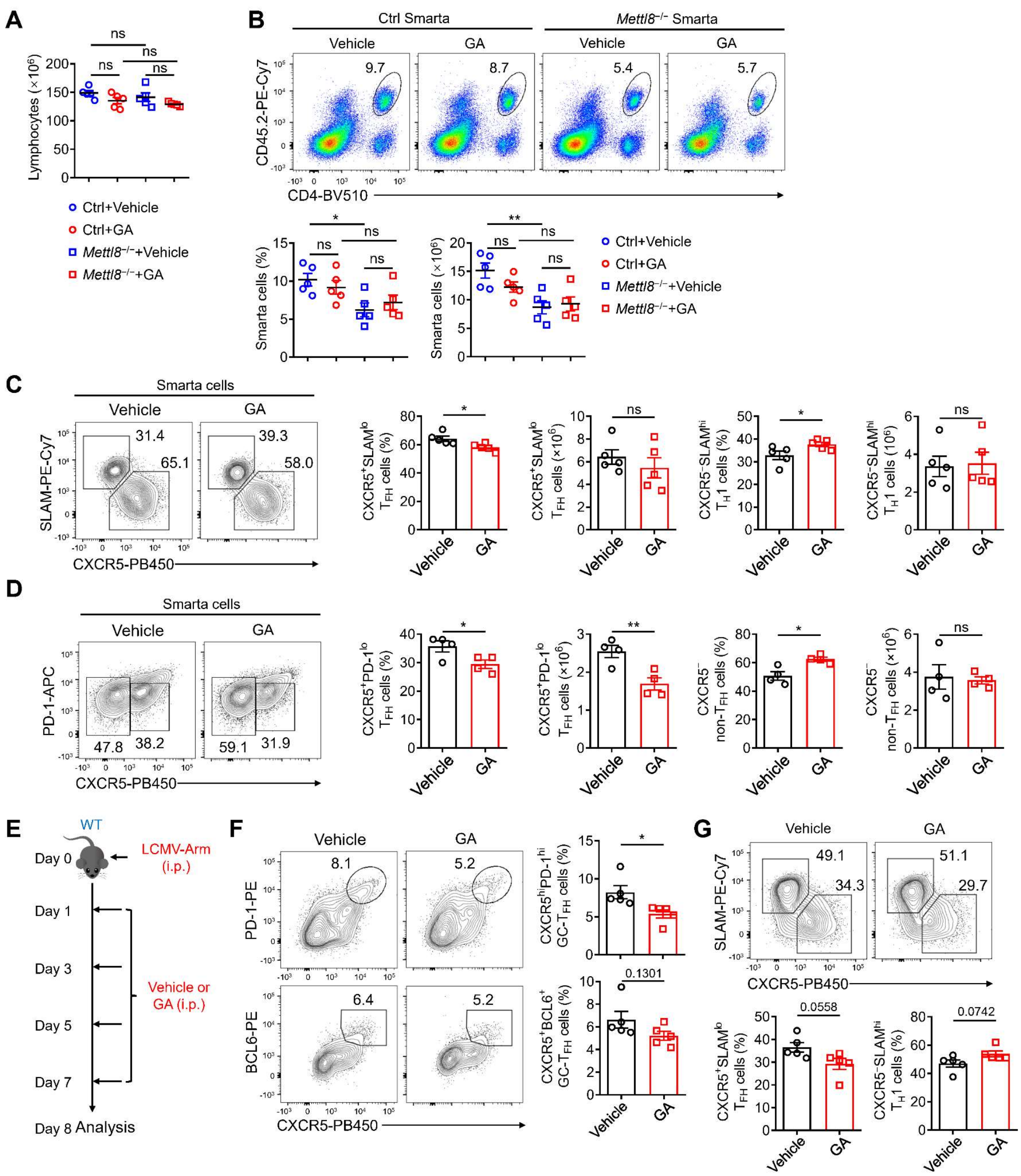

Supplemental Figure 9. Suppression of T$_{FH}$ cell differentiation by ginkgolic acid (GA). (**A** and **B**) In the adoptive transfer model, absolute numbers of lymphocytes (**A**) and representative plots of CD45.2$^+$ donor cells in recipient spleens were (**B**) recorded (n = 5/group). (**C** and **D**) SLAM^{lo} CXCR5$^+$ T$_{FH}$ and SLAM^{hi} CXCR5$^-$ T$_H$1 cells (**C**, n = 5/group), and PD-1^{lo} CXCR5$^+$ T$_{FH}$ and CXCR5$^-$ non-T$_{FH}$ cells (**D**, n = 4/group) in CD45.1$^+$ Smarta CD4$^+$ T cells were illustrated in representative plots, proportions, and absolute numbers. (**E**) Ginkgolic acid-treated directly infected model was schematically illustrated. (**F**−**G**) Representative plots and quantification of PD-1^{hi} CXCR5^{hi} and

BCL6$^+$ CXCR5$^+$ GC T_{FH} cells (**F**), and SLAM^{lo} CXCR5$^+$ T_{FH} and SLAM^{hi} CXCR5$^-$ T_H1 cells in the directly infected model (n = 5/group). Data represent 2–3 independent experiments, mean ± SEM. P values were calculated using one-way ANOVA (**A** and **B**), and two-tailed Student's *t*-test (**C**, **D**, **F**, and **G**), $^*p < 0.05$; $^{**}p < 0.01$.

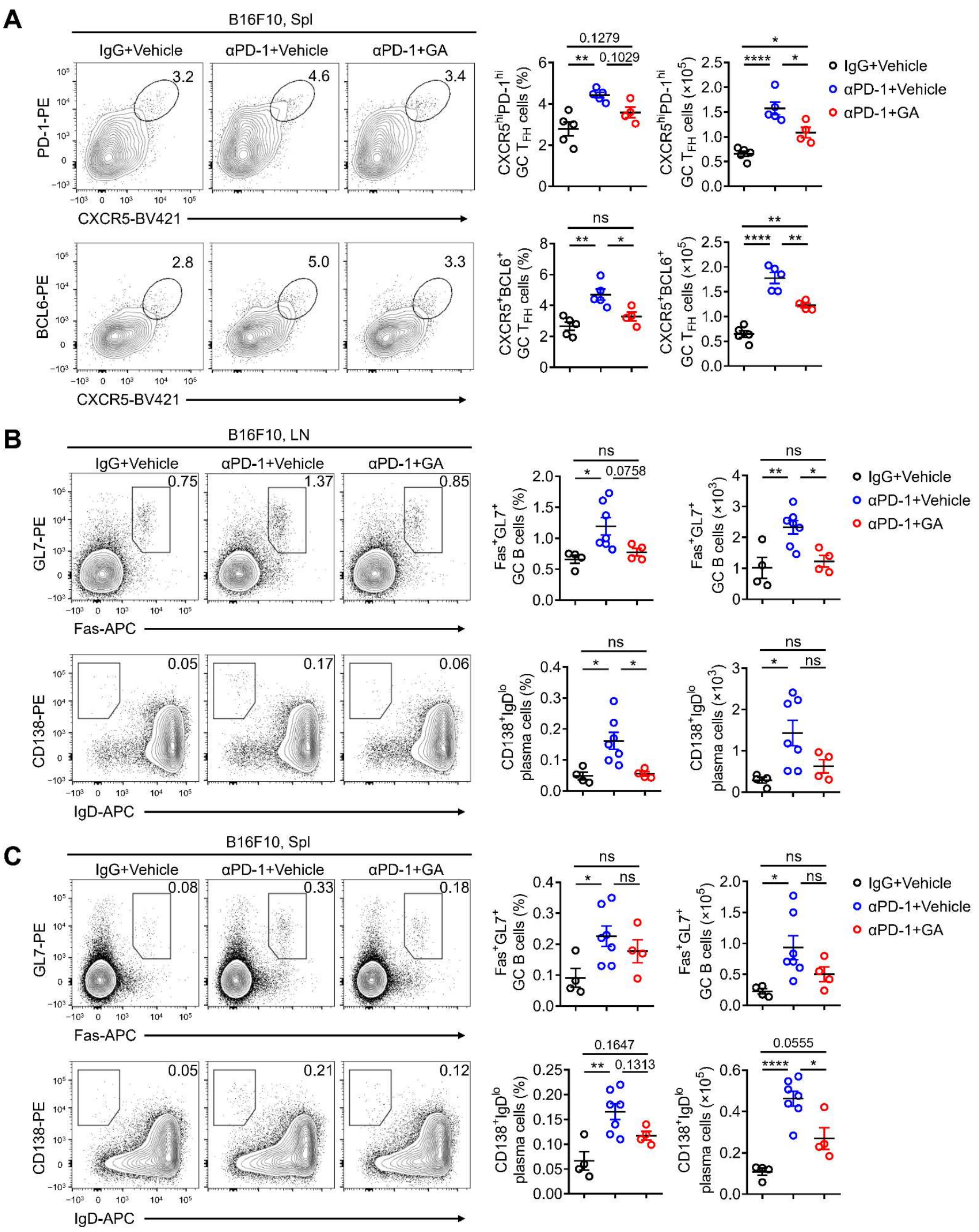

Supplemental Figure 10. Suppression of T_{FH}-associated B cell responses by GA treatment. (**A**) Representative plots (left) and statistical analysis (right) showing PD-1^{hi} CXCR5^{hi} and BCL6^{+} CXCR5^{+} GC T_{FH} cells gated on CD44^{hi} CD62L^{lo} CD4^{+} T cells in the spleens of B16F10 tumor-bearing mice treated with IgG, anti-PD-1 alone, or anti-PD-1 plus GA (n = 4–5/group). (**B** and **C**) Representative plots (left) and statistical analysis (right) showing GL-7^{+} Fas^{+} GC B cells and CD138^{+} IgD^{lo} plasma cells gated on B220^{+} CD19^{+} cells in the TdLNs (**B**) and spleens (**C**) of B16F10 tumor-bearing mice treated with IgG, anti-PD-1 alone, or anti-PD-1 plus GA (n = 4–7/group). Data

represent 2–3 independent experiments, mean ± SEM. P values were calculated using one-way ANOVA, *$p < 0.05$; **$p < 0.01$; ****$p < 0.0001$.

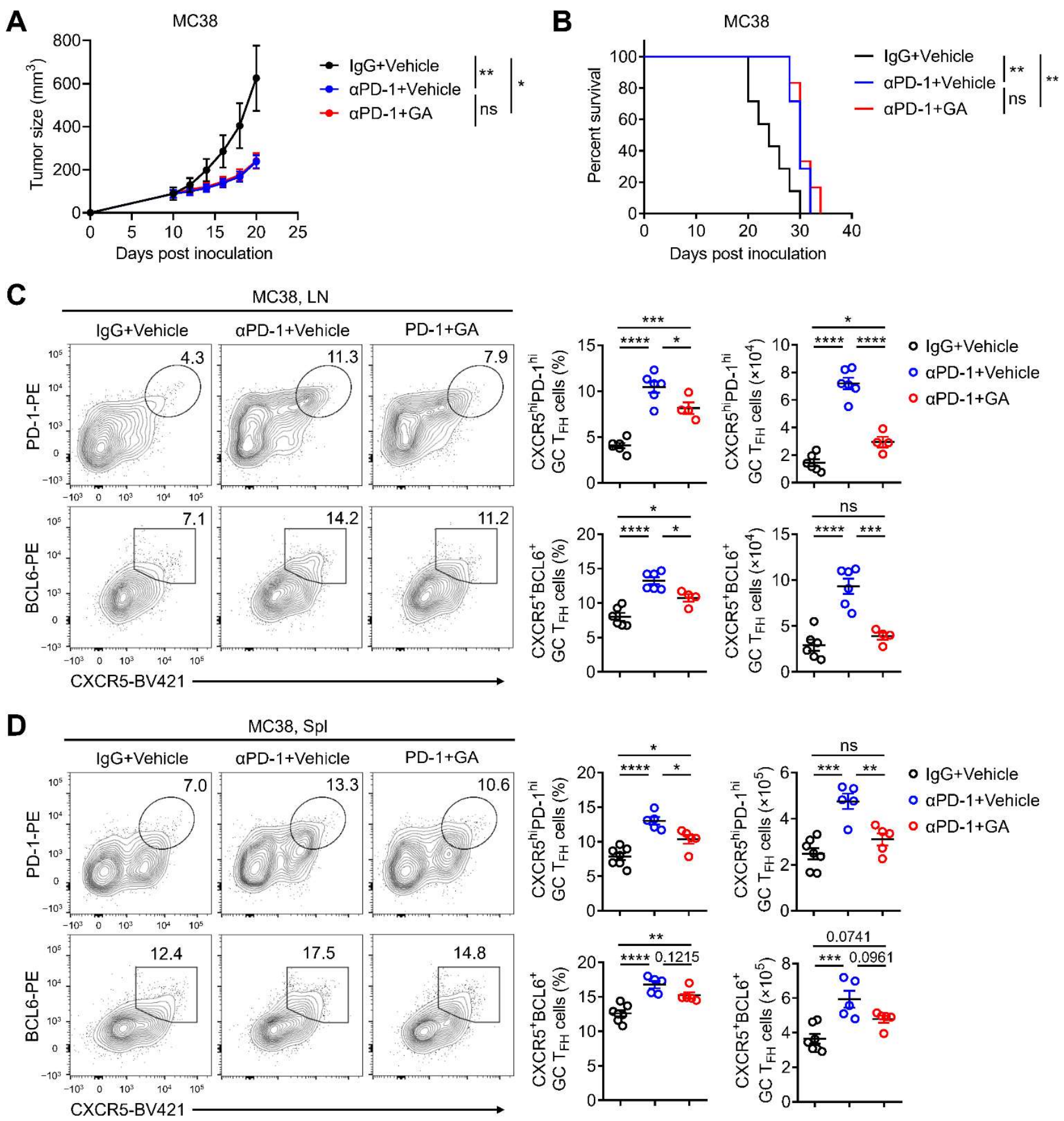

Supplemental Figure 11. GA suppresses T_{FH} cell differentiation in the MC38 tumor model. (**A** and **B**) Tumor growth (**A**) and survival curve (**B**) of MC38 tumor-bearing mice treated with IgG, anti-PD-1 alone, or anti-PD-1 plus GA (n = 7/group). (**C** and **D**) Representative plots (left) and statistical analysis (right) showing PD-1^{hi} CXCR5^{hi} and BCL6^{+} CXCR5^{+} GC T_{FH} cells gated on CD44^{hi} CD62L^{lo} CD4^{+} T cells in the TdLNs (**C**, n = 4–6/group) and spleens (**D**, n = 5–7/group) of MC38 tumor-bearing mice treated with IgG, anti-PD-1 alone, or anti-PD-1 plus GA. Data represent 2–3 independent experiments, mean ± SEM. P values were calculated using the two-way ANOVA (**A**), Log-rank test (**B**), and one-way ANOVA (**C** and **D**), *$p < 0.05$; **$p < 0.01$; ***$p < 0.001$; ****$p < 0.0001$.
