# Supplementary material for: METTL8 orchestrates TFH cell differentiation through m³C-mediated TCF1 regulation in infection and cancer models
Source: J Clin Invest. 2026 Nov 16;136(22):e199747. doi: 10.1172/JCI199747 (PMC13767623; doi:10.1172/JCI199747)

Full unedited blot for Figure 6G

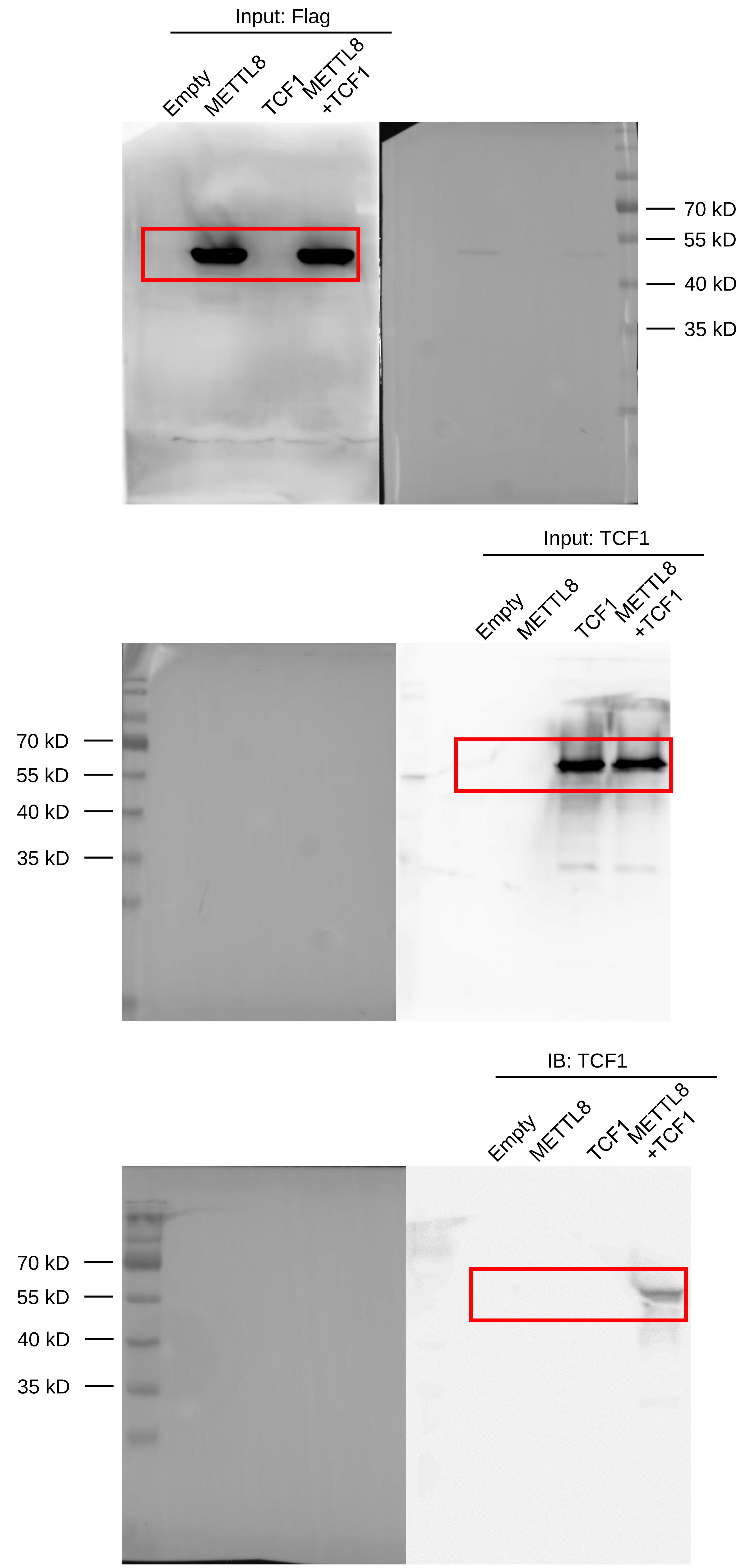

Full unedited blot for Figure S6A

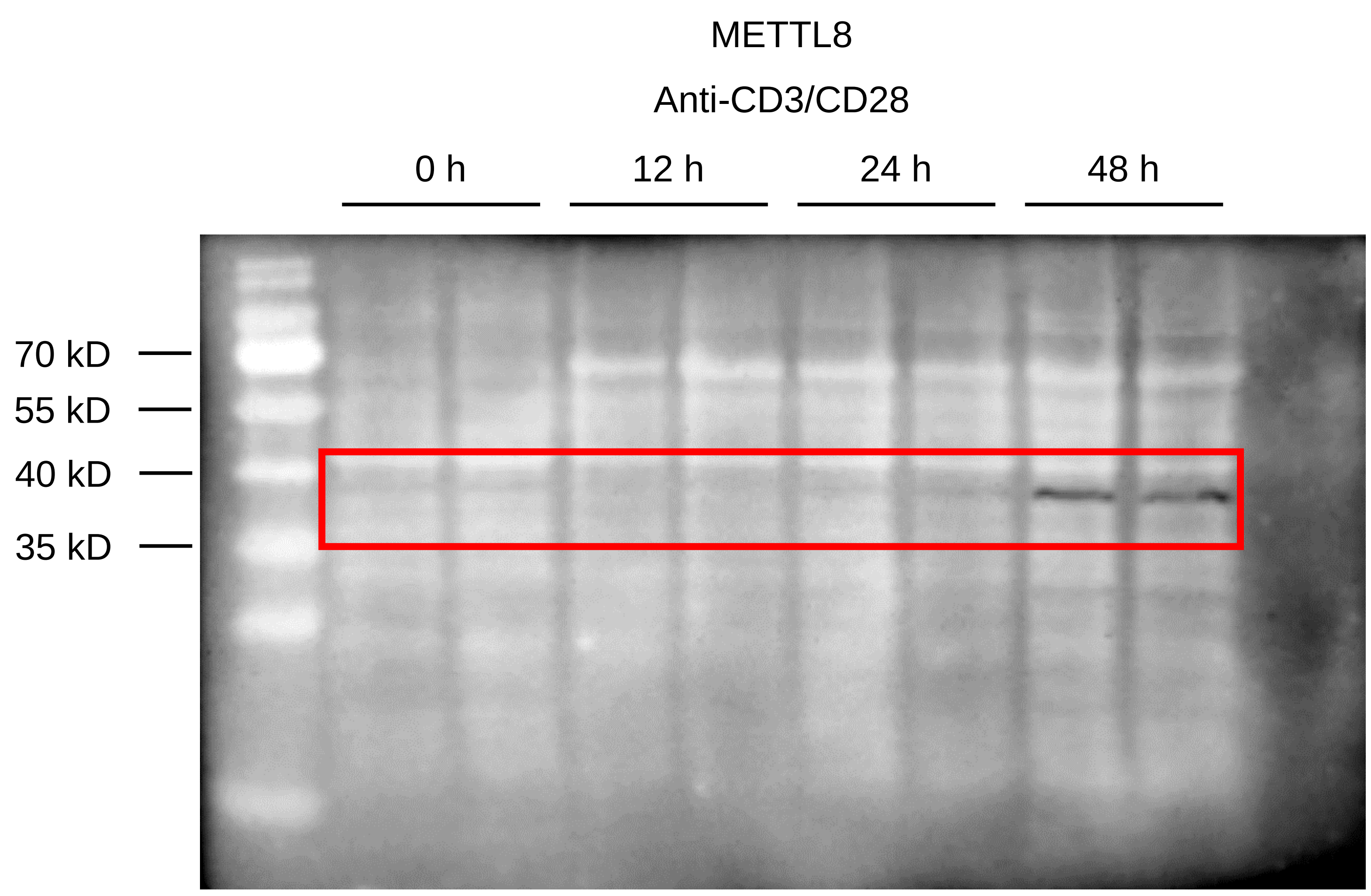

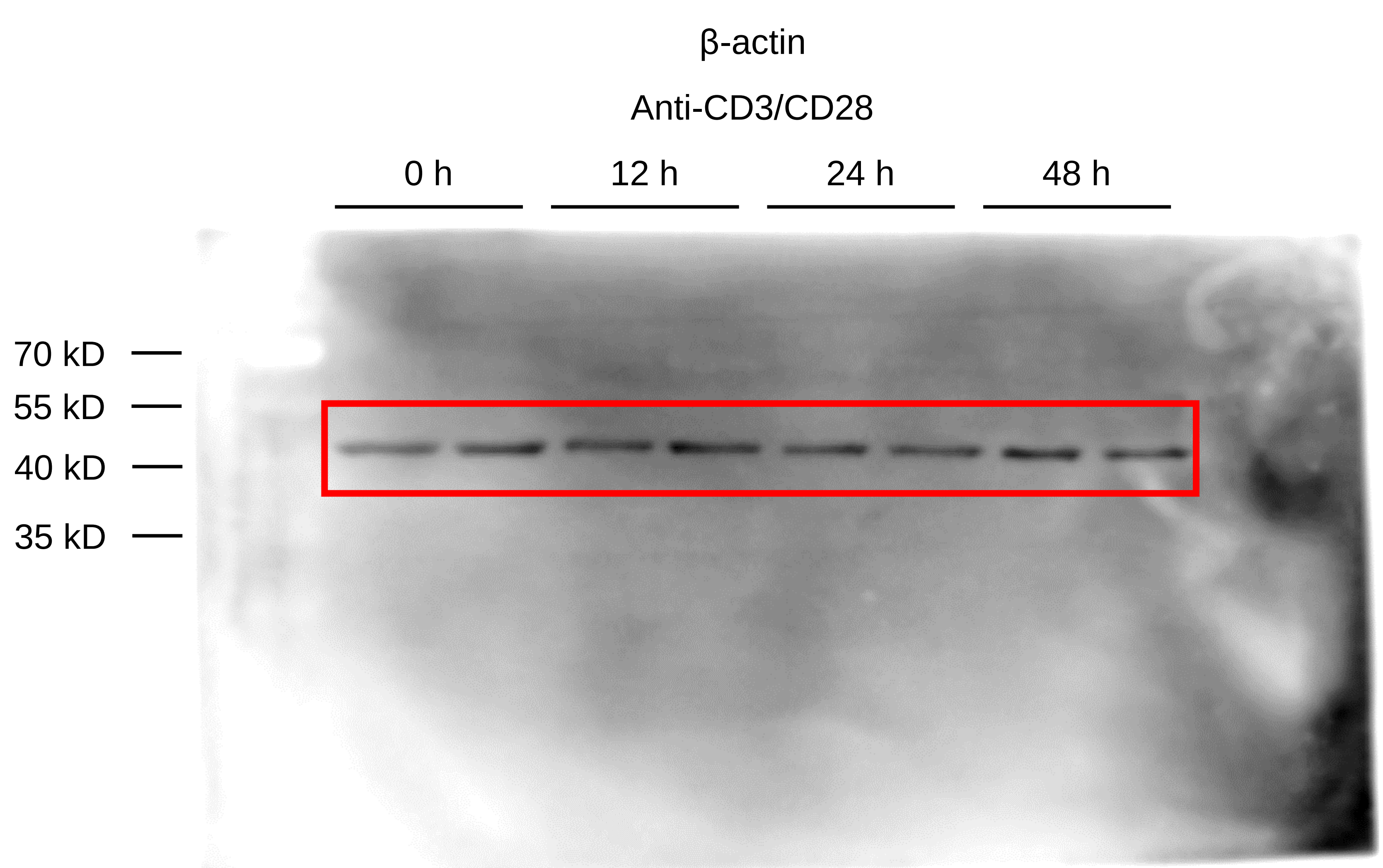

Supplement: Supplementary file 2 [file jci-136-199747-s081.pdf]
